# Recent Advances in Pharmaceutical Approaches of Antimicrobial Agents for Selective Delivery in Various Administration Routes

**DOI:** 10.3390/antibiotics12050822

**Published:** 2023-04-27

**Authors:** Ardiyah Nurul Fitri Marzaman, Tri Puspita Roska, Sartini Sartini, Rifka Nurul Utami, Sulistiawati Sulistiawati, Cindy Kristina Enggi, Marianti A. Manggau, Latifah Rahman, Venkatram Prasad Shastri, Andi Dian Permana

**Affiliations:** 1 Faculty of Pharmacy, Hasanuddin University, Makassar 90245, Indonesia; marzamananf21n@student.unhas.ac.id (A.N.F.M.); roskatp21n@student.unhas.ac.id (T.P.R.); sartini@unhas.ac.id (S.S.); rifkanurulutami@unhas.ac.id (R.N.U.); sulistiawati18n@student.unhas.ac.id (S.S.); enggick18n@student.unhas.ac.id (C.K.E.); mariantimanggau@unhas.ac.id (M.A.M.); latifahrahman@unhas.ac.id (L.R.); 2 Institute for Macromolecular Chemistry, Albert Ludwigs Universitat Freiburg, 79085 Freiburg, Germany; prasad.shastri@gmail.com

**Keywords:** antimicrobial resistance, antimicrobials, pharmaceutical preparations, selective delivery

## Abstract

Globally, the increase of pathogenic bacteria with antibiotic-resistant characteristics has become a critical challenge in medical treatment. The misuse of conventional antibiotics to treat an infectious disease often results in increased resistance and a scarcity of effective antimicrobials to be used in the future against the organisms. Here, we discuss the rise of antimicrobial resistance (AMR) and the need to combat it through the discovery of new synthetic or naturally occurring antibacterial compounds, as well as insights into the application of various drug delivery approaches delivered via various routes compared to conventional delivery systems. AMR-related infectious diseases are also discussed, as is the efficiency of various delivery systems. Future considerations in developing highly effective antimicrobial delivery devices to address antibiotic resistance are also presented here, especially on the smart delivery system of antibiotics.

## 1. Introduction

Globally, the top three causes of death are cardiovascular (ischemic heart disease and stroke), respiratory (chronic obstructive pulmonary disease and lower respiratory infections), and neonatal conditions (birth asphyxia, birth trauma, neonatal sepsis and infections, and preterm birth complications). According to WHO data in 2020, lower respiratory infections were the fourth leading cause of death worldwide. In 2019, 2.6 million people died, 460,000 fewer than in 2000. The threat posed by infectious diseases necessitates research into effective responses to medication.

Since they were discovered early in the 1920s and were introduced in the 1940s, antibiotics have played an important role in eradicating infectious diseases [1]. Antibiotics inhibit cell wall or protein synthesis, damage bacterial membranes, cause interior substance loss, and more [2]. However, several problems related to antibiotics continue to rise and become a limitation in the effective treatment of infectious diseases. Antimicrobial resistance (AMR) has been observed as a natural occurrence ever since penicillin was first used in the 1940s. When clinically significant resistance has developed, it has been met with either the introduction of new antibiotic classes or the modification of existing antibiotic classes that have low rates of cross-resistance [3]. AMR is a significant threat worldwide. Recent figures show that 1.2 million deaths were caused by resistant bacterial infections in 2019 and 4.95 million in 2020 [4].

A few approaches to narrower-spectrum antibiotics and rapid diagnostics are being discussed, as are the theoretical benefits and drawbacks of this new approach to bacterial infectious diseases [5]. Targeting the pathogen and delivering a toxic payload creates two resistance pathways. The use of antibodies or bacteriophages to fight the pathogen can be another strategy that is well-documented [5]. However, during the process, a lack of specific targeting of antibiotics leads to low efficacy caused by inadequate drug concentration at sites of infection [6,7], in addition to off-target effects such as increased adverse toxicity towards healthy cells [8]. Long-term and inappropriate use of antibiotics has led to the development of antimicrobial resistance (AMR) [9,10,11]. It may be possible to solve these challenges by designing a customized and responsive drug delivery system so that more of the drug is delivered directly to the infection site. Drug targeting helps conjugates bind specifically to the pathogen and create a locally high antibiotic concentration that kills the target. This strategy theoretically protects non-targeted bacteria (and host cells for general toxins) from toxic payload concentrations, which means less drug components will accumulate in healthy tissue and there is a lower risk of side effects [5,12].

Internal stimuli (pH, temperature, enzyme, redox), as well as external stimuli (light, magnetic, ultrasound), are both viable options for designing a targeted and responsive drug delivery system. It is well known that the microenvironment of an infection site has different characteristics. Infection sites tend to have low pH due to anaerobic glycolysis which in turn results in local accumulation of lactic and acetic acid. This characteristic can be used as an approach for targeted drug delivery by pH stimuli. Using the fact that bacterial cell walls are negatively charged, it can be utilized as another option to directly deliver antibiotics to infected tissue [13]. For example, Zhao et al. (2019) established cationic-charged polymers for the delivery of chlorhexidine in the oral cavity under an acidic environment. Cariogenic biofilm (pH value around 5.5) could change to positively charged from negatively charged caused by the degradation of the citraconic amide group, followed by the release of chlorhexidine at the infection site. In addition to that, the bacterial infection microenvironment also exhibits different redox potential compared to normal cells, which is controlled and determined by levels of Nicotinamide adenine dinucleotide phosphate (NADPH/NADP^+^) and glutathione [14]. This way could provide an opportunity for the development of redox-responsive drug delivery systems [15,16]. A temperature responsive of Chloramphenicol has been developed to have a good antibacterial effect with 99.95% eradication of *Staphylococcus aureus* bacteria on infected skin model through ex vivo evaluations [17]. Bacterial infection is also associated with (or accompanied by) increased secretion of enzymes such as hyaluronidase and chymotrypsin. These enzymes could potentially develop as a target in delivering antibiotics. Yao et al. (2017) signed and constructed an enzymatically degradable composite of multilayer films (hyaluronic acid/chitosan)n-(hyaluronic acid/polylysine)n, also known as (HA/CHI)n-(HA/PLL)n, which led to an enzymatic breakdown of the multilayer films and efficiently reduced the adherence of both *Staphylococcus aureus* and *Escherichia coli* bacteria (>99%) after 24 h [18].

The possibility of bacteria to create biofilms by forming physical barriers contributes more to the mechanism of resistance. Biofilms are bacterial colonies embedded in a matrix of extracellular polymeric substances (EPS), which includes lipids, proteins, polyols, and even genetic material from the host cell. The existence of biofilm makes gene transfer more likely to occur, and it also contributes to the spread of antimicrobial resistance. Gebreyohannes et al. (2019) reported that the biofilms of bacteria exhibit 10- to 1000-fold higher resistance to the treatment of antibiotics [19]. Drug delivery systems that are responsive to the biofilms’ microenvironment would be a promising way to overcome this problem. The development of nanoparticles loaded with deoxyribonuclease (DNase) and protease was an example of new approaches due to the presence of biofilms that targeted the biofilm structure to improve penetration of antibacterial drugs [1,7,20,21].

The external stimulus could also be exploited as an element in the design of selectively responsive drug delivery systems [8,9,12]. Light-responsive biomaterials have shown promise as a non-invasive, highly-precise tool for the controlled release of medicines and other therapeutic compounds [22,23]. In addition to that, the use of magnetic stimulation as the drug delivery system, could possibly monitor drug concentration and distribution, and impact drug release, allowing precise control over drug delivery targeted area [24]. These products comprise biocompatible Fe3O4 superparamagnetic iron oxide nanoparticles (SPIONs) [24]. The exploration of light and magnetic field as an external stimuli for the localized, controlled delivery of drugs are developing [25]. Patel et al. (2021) created a combinational antibiotic and photothermal (PT) therapy to kill bacteria between targeted near-infrared (NIR) laser-activated drug delivery nano-assemblies and PT therapy (PTT) [26]. Both treatments work better together by protecting antibiotic delivery and release near the bacteria surface and lower off-target toxicity. Using the ultrasound release mechanism, Wu et al. found that pulsed-laser irradiation of liposomes containing hollow gold nanoshells (HGNs) or mixed with free HGNs caused the release of encapsulated 6-carboxyfluorescein (CF), a fluorescent dye, above a threshold of 1.5 W cm^2^ without causing permanent liposomal damage or significant increases in bulk solution temperature [27]. The use of superparamagnetic iron oxide nanoparticles showed that magnetism could trigger the release of antibiotics from drug-delivery vehicles from the co-encapsulation of methicillin within the aqueous core of iron oxide polymersomes (IOPs). This approach demonstrated efficacy in eradicating biofilms [28].

In addition, a wide variety of different delivery systems have been created to localize the distribution of antimicrobial medicines to locations of infection throughout the body. This review provides an overview of common infection sites and the diseases developed as a consequence. Furthermore, the available conventional therapy and drawbacks behind it are discussed and we summarized current research conducted in the field on specific and responsive targeted drug delivering systems for infectious diseases, either based on internal or external stimulus for the eradication of microorganisms, which contributes to infection and antimicrobial resistance. It is necessary to limit the continued development of AMR by using an advanced drug delivery system to selectively release antimicrobials drug into the target tissue, while at the same time being able to promote the rational use of antimicrobials.

## 2. Sites of Infection

### 2.1. Skin and Soft Tissue Infections

Skin and soft tissue infections (SSTIs) are one of the largest groups of infectious diseases. The skin is the largest and outermost organ of the body and serves as the main protective barrier from environmental exposure and foreign objects. In fact, the surface of the skin is colonized by various classes of microorganisms, both bacteria and fungi, which have come to be known as normal skin flora. Most of these microorganisms are harmless and even able to compete with pathogenic microorganisms. Alterations in skin conditions due to trauma or medical intervention can allow bacteria to penetrate beneath the surface, which may lead to infection [29,30]. SSTIs include infections that occur on the surface of the skin, the fascia layer, subcutaneous fat, and the underlying muscles and tendons. Diabetes mellitus and immunosuppression are also predisposing factors with high risk. Because of the variety of infectious agents causing risk factors in patients, the symptoms of SSTIs vary from mild to life-threatening. In particular, *Staphylococcus aureus* and group A streptococci, which are Gram-positive bacteria, are considered to be the main causes of SSTIs. It was reported that more than half of SSTI cases referred to the emergency department were caused by community-acquired MRSA (methicillin-resistant *Staphylococcus aureus*). However, SSTIs can also be caused by Gram-negative bacteria, fungi, viruses, and parasites [31,32].

In general, SSTIs can be classified according to their complications and purulence. Non-complicated infections affecting the skin and superficial soft tissues can be treated without hospitalization. Examples of these conditions include impetigo, erysipelas, cellulitis, folliculitis, abscesses, and trauma-related infections. These infections are also called superficial SSTIs because they only occur on the surface layer of the skin, not deeper than the subcutaneous fat layer [31,33].

Complicated SSTIs are divided into necrotizing and non-necrotizing. Complicated infections require inpatient treatment consisting of, for example, deep abscesses, Fournier’s gangrene, necrotic fasciitis, and infections caused by human or animal bites. In addition, SSTIs were divided into mild, moderate, and severe based on their purulence [30,34]. The classification system of SSTIs based on severity and the presence of comorbidities is very important in determining the next steps to be taken in medical intervention.

### 2.2. Mucosal Layer Infections

This section covers infections that commonly occur in several parts of the body that are protected by mucosal layers. This section will discuss the common infections that occur in the eye and vagina, while rectal infections are discussed in the section titled gastrointestinal tract. However, due to the anatomical and physiological differences between these organs, special attention must be paid to the type of infection that may be found.

In the case of eye infections, the usual symptoms are red eyes, local pain, and blurred vision. Conjunctivitis, which can be caused by viruses or bacteria, is the most frequent type of ocular infection. This condition is a mild condition that is usually characterized by red, painful eyes with discharge and can be treated easily. However, it is highly contagious. Viral conjunctivitis is more common, and nearly 90% of cases are caused by adenovirus. For rarer cases, herpes simplex or zoster virus may be involved [35]. On the other hand, bacterial conjunctivitis is more common in children. The causative bacteria include *Staphylococcus aureus*, *Streptococcus pneumoniae*, and *Haemophilus influenza* [36]. More serious eye infections can be exemplified by infectious keratitis and endophthalmitis. The main predisposing factor in infectious keratitis is frequent contact lens use. This condition is also known as corneal ulceration and is a major cause of ocular morbidity. The spread of the epidemiology of this disease is highly dependent on the region, with bacterial infections more common in temperate climates (or regions) than tropical regions [37]. Bacteria frequently associated with corneal ulceration include *Staphylococcus aureus*, *Pseudomonas aeruginosa*, Enterobacteriaceae, and *Streptococcus pneumonia* [38]. Some fungal species are also the cause of keratitis, especially in the tropics. Meanwhile, endophthalmitis is an infection of the deeper part of the eye. Although rare, this condition is often irreversible and can cause permanent blindness. The cause of this infection is generally Gram-negative bacteria, such as *Klebsiella pneumoniae*, *Pseudomonas aeruginosa*, and a group of Enterobacter bacteria [39].

Another layer of mucosa that is susceptible to infection is the female genital tract. The term vaginitis is usually used to describe several types of infections that occur in the vagina. This organ originally has a normal flora to maintain pH and inhibit the growth of pathogenic microorganisms. However, changes in conditions in the growth of normal flora will disrupt the balance. Vaginal infections can be caused by bacteria (bacterial vaginosis, BV), fungi (vulvovaginal candidiasis, VVC), or protozoa (trichomoniasis). Although there are different causes, this condition shows almost similar symptoms, including abnormal discharge and an unpleasant odor, a burning or itching sensation, and irritation [40,41]. In BV, the environment that was initially dominated by the normal flora Lactobacillus is replaced by a mixture of facultative and anaerobic polymicrobial, including Mycoplasma, Ureaplasma, Streptococcus, Bacteroides, Gardnerella, and Mobiluncus, etc. [42]. Meanwhile, VVC, also known as vaginal thrush, is caused by Candida species, generally C. albicans. However, in recent years, this has also been found to be caused by other species such as *C. tropicalis*, *C. glabrata*, or *C. krusei* [43]. VVC that is not treated properly can develop into recurrent cases, namely a condition in which a woman experiences more than four episodes per annum [44].

### 2.3. Respiratory Tract Infections

Respiratory tract infections can be distinguished based on their location, namely upper and lower respiratory tract infections (URTIs and LRTIs). The upper respiratory tract includes the nose, pharynx, larynx, and larger airways. Both viruses and bacteria can cause URTIs. Associated conditions are the common cold, influenza, laryngitis, tonsillitis, acute rhinitis, and acute otitis media [45,46]. Diagnosis is generally difficult in patients with URTIs because of similar and overlapping symptoms. Infection of the upper airways can also cause a non-pneumonic cough but is generally not life-threatening. Symptoms may become self-limiting, and the patient may require bed rest, but they may resolve on their own. Most viral URTIs are caused by rhinoviruses. Other viral causes include influenza and parainfluenza virus, coronavirus, adenovirus, and respiratory syncytial virus (RSV) [47]. On the other hand, URTIs can also be caused by bacteria, such as *Haemophilus influenzae*, *Staphylococcus aureus*, *Streptococcus pneumoniae*, and *Moraxella catarrhalis* [48].

Compared to URTIs, LRTIs represent a more severe condition and often require hospitalization. These infections are considered to be one of the leading causes of death worldwide. LRTIs include conditions such as acute bronchitis, bronchiolitis, tracheitis, pneumonia, and other infections of the lungs caused by bacteria, fungi, viruses, and, in isolated cases, parasites [45,49]. Prior to the discovery of antibiotics, *Streptococcus pneumoniae* was the etiology of community-acquired pneumonia (CAP), with an incidence exceeding 90%. However, nowadays, with advancements in antimicrobial therapy and vaccination, some statistics report that nearly 2/3 of CAP is caused by viral infection. Viral and bacterial co-infections have also been reported, with an incidence of up to 7%. Viral infection of the lower airways can reduce mucociliary clearance which can promote bacterial colonization. Other bacteria that are also associated with CAP are *Haemophilus influenzae* and *Pseudomonas aeruginosa*. Rhinovirus and influenza virus types A and B are the main viral causes of CAP [50,51]. Higher mortality rates were reported in cases of viral and bacterial co-infections [52]. In addition, nosocomial pneumonia can also occur, also known as hospital-associated pneumonia (HAP). In contrast to CAP, HAP is mostly caused by bacteria, including *Streptococcus aureus*, *Pseudomonas aeruginosa*, and *Klebsiella pneumonia* [49,53]. Establishing an appropriate etiologic diagnosis of pneumonia is necessary for decision-making in the management and selection of pharmacotherapy.

Pulmonary tuberculosis (TB) is another type of lung infection caused by Mycobacterium tuberculosis. This disease is considered a global problem because of the number of deaths it causes, especially in developing countries. The spread of this disease occurs through the air. After containment of the bacteria in the host, also known as latent TB, symptoms will appear, and thus the patient will enter the contagious state. Symptoms commonly found in active TB include persistent cough, fever, night sweats, and even weight loss. TB can also affect other organs in the body, and its clinical manifestations can vary from mild to life-threatening [54,55,56].

### 2.4. Gastrointestinal Infections

Gastrointestinal (GI) tract infections are still a health burden that requires special attention. Although its prevalence has decreased significantly in Western countries due to improved sanitation and advancements in food technology, this water-borne disease is still common in low-income and under-developed societies and presents a higher risk in immunocompromised patients [57]. In addition to food-borne transmission, transmission can also occur between humans or upon contact with environmental factors such as stagnated water and human excrement. The most common clinical manifestation of GI tract infection is diarrhea, which can be acute (<7 days), prolonged (7–14 days), persistent (14–29 days), and chronic (>30 days) [58]. Other symptoms that may follow are nausea/vomiting, fever, and abdominal pain. A bacterial peptic ulcer caused by Helicobacter pylori is an infection that occurs in the upper GI tract. Careful diagnosis must be maintained to differentiate it from an NSAID-related peptic ulcer. This infection affects the gastric lining to the upper part of the duodenum and can cause GI bleeding [59,60] and also increase the likelihood of cancer.

Infections of the lower GI tract on the other hand can generally lead to chronic conditions, for example, enteric infection, a condition called irritable bowel syndrome (IBS), is characterized by persistent GI symptoms. Infectious IBS must be differentiated from idiopathic conditions such as Crohn’s disease or ulcerative colitis. The most common causative microbes are Salmonella, Shigella, Clostridium, and Campylobacter from the bacterial group, Candida from the yeast family, and parasites such as Entamoeba histolytica, Giardia lamblia, cryptosporidium, and Cyclospora. Determination of specific causative organisms is imperative in the diagnosis and management of this disease [61,62].

### 2.5. Urinary Tract Infections (UTI)

Urinary tract infections (UTIs) constitute a major fraction of infectious diseases with 150 million cases per year [63]. Due to their high rate of morbidity, UTIs also present a significant economic burden due to hospitalization and sick days. UTIs without additional compromise of the host urinary tract are referred to as uncomplicated (uUTIs). Based on their location, uUTIs that occur in the urethra and bladder are called cystitis, while those that occur in the upper urinary organs are termed pyelonephritis. Uncomplicated cases can be resolved with the use of appropriate antibiotics [64]. On the other hand, if the patient has nephrological disorders or is pregnant, there is a high risk of infection occurring in the form of complicated UTIs (cUTIs). Uropathogenic *Escherichia coli* (UPEC) is the leading cause, either uncomplicated or complicated, and is reported to account for about 80% of the cases. Several other species of microorganisms have also been associated with UTIs, such as Enterococcus faecalis, group B streptococcus, Klebsiella pneumoniae, and, in rare cases, Candida spp. Risk factors for uUTIs include female gender, sexual activity, vaginal infections, history of UTIs, diabetes, and obesity. For cUTIs, approximately 80% of cases are associated with the use of an indwelling catheter [65,66].

### 2.6. Lymphatic Infections

The lymphatic vascular system’s primary function is maintaining tissue fluid balance and transporting immune components within the body [67]. Although not the primary circulatory system in higher animals such as the vertebrae, the lymphatic system has received particular attention in the last decades because of its role in various diseases. Lymphadenopathy is a general term used to refer to a group of diseases related to the lymph nodes; however, it is not limited to infectious diseases and is characterized by swollen lymph nodes. When it is due to bacteria-induced inflammation it is known as lymphadenitis. The most commonly associated bacteria are *Streptococcus pyogenes* and *Staphylococcus aureus*. Meanwhile, lymphangitis is a more severe inflammation of the lymphatic vasculature system in the subcutaneous layer involving bacteria (acute), or in more chronic conditions it can be caused by fungi, mycobacterium, or parasites [68,69,70]. In tropical regions, some parasites such as *Wuchereria bancrofti*, *Brugia timori*, and *Brugia malayi* can invade the lymphatic system. This invasion leads to a disease called lymphatic filariasis and can cause permanent disability in patients [71,72].

### 2.7. Central Nervous System (CNS) Infections

CNS infection is a serious condition with very high morbidity and mortality rates and often requires neurosurgical intervention. Most CNS infections are caused by bacteria and viruses, but fungal infections have also been reported [73,74]. Meningitis, an infection of the meninges and subarachnoid space, can result from direct exposure from the community (community-acquired) or from hospitalization (nosocomial infection). The most common pathogens in community-acquired cases include *Streptococcus pneumoniae*, *Listeria monocytogenes*, and *Neisseria meningitidis* [75]. Having a slightly lower mortality rate, meningitis can also be caused by viral infections, including *enterovirus*, *herpes simplex*, and *varicella zoster*. Confirmation of the pathogen is done by detection in the cerebrospinal fluid [76]. Encephalitis is the inflammation of the brain parenchyma caused by infection. Many agents, including bacteria, viruses, fungi, and protozoa, can lead to this condition. Neuroimaging is useful in etiological diagnosis. The incidence of viral encephalitis is greater when compared to bacterial [77,78]. Some other less common intracranial CNS infections include ventriculitis and CNS tuberculosis [79].

## 3. Commonly Used Antimicrobials for the Treatment of Infectious Diseases Caused by Microorganisms

### 3.1. Skin Infection

#### 3.1.1. Bacterial

When it comes to opportunistic infections caused by *Pseudomonas aeruginosa*, skin infection is one of the most common. The studies of empirical antibiotics have established them to be an option to treat *Pseudomonas aeruginosa* skin infections. Currently, *β-lactams*, clindamycin, and fluoroquinolones are recommended for mild to severe infection involving bacteria of the *Pseudomonas aeruginosa* family [80,81]. In the treatment of *P. aeruginosa* skin infection, *β-lactams* are the most frequently used therapeutic agent administered orally and intravenously for severe cases as well as fluoroquinolones to treat both Gram-positive and Gram-negative bacteria, making them ideal for the treatment of infection caused by *Pseudomonas aeruginosa*. The use of combined therapy, such as amikacin, ceftazidime, imipenem, and ciprofloxacin, is also indicated to achieve a synergistic effect [82]. The antibacterial mechanism of β-lactams lies in its ability to hinder the formation of bacterial cell walls by binding to the penicillin-binding proteins (PBPs). Penicillin and its semi-synthetic β-lactam analogues amoxicillin and their combination with β-lactamases inhibitors (clavulanate acid and sulbactam) have been used to treat MRSA infection [83]. Furthermore, clindamycin is another option for treating *Pseudomonas aeruginosa* skin infection due to its activity against several Gram-positive bacteria [84]. Additionally, vancomycin and piperacillin/tazobactam are first-line treatments for severe nonpurulent infections, especially suspected necrotizing or polymicrobial infections. This combination increases the risk of acute renal injury compared to vancomycin alone or with other beta-lactams [85]. Combination therapy demonstrates a significant benefit against the pathogen [33]. In the case of MRSA, bactericidal antibiotics such as vancomycin or daptomycin should be added to first-line therapy [86,87].

*Streptococcus pyogenes*, *Vibrio vulnificus*, or *Aeromonas hydrophila* may induce necrosis of the skin in necrotizing fasciitis. For necrotizing infections, clindamycin and penicillin should be administered intravenously. It is possible to treat mixed necrotizing infections with an array of antimicrobials targeted against aerobic Gram-positive and Gram-negative bacteria as well as anaerobes. Infection caused by *Pasteurella multocida* is known to be resistant to dicloxacillin, cephalexin, erythromycin, and clindamycin. Therefore, people with moderate penicillin allergies could receive parenteral cefoxitin or carbapenem antibiotics [88]. Group of oxazolidinones known as linezolid had the ability to kill MRSA and macrolide-resistant streptococci which can be administered in IV and oral. Patients with renal impairment can switch to oral linezolid without dose decrease. Nausea and vomiting occur most often. Tigecycline has caused acute pancreatitis [89]. *Staphylococcus aureus* and/or *Streptococcus pyrogenes* infections have been linked to impetigo, a skin illness. However, older children and adults might also be affected [90]. Impetigo can be treated on the face, eyelid, or mouth, depending on the site of the illness. Although resistance to mupirocin has been reported, it remains the best topical treatment option [91]. Bacitracin and neomycin, on the other hand, are other options, but less effective therapies [88,90].

Numerous skin-specific or niche-specific microorganisms have been implicated in cellulitis, an illness caused by *Staphylococcus aureus*. Penicillin is the drug of choice for treating cellulitis, whether administered intravenously or orally, depending on the severity of the infection. Anti-staphylococcal drugs may have a shorter half-life in patients with cystic fibrosis, obesity, kidney disease, or in young children [84,86]. Penicillinase-resistant or semi-synthetic penicillin or another first-generation cephalosporin should be recommended for cellulitis [88,90]. MRSA-related skin infection are rarely treated with fluoroquinolones like ciprofloxacin due to MRSA’s lower susceptibility to fluoroquinolones. Delafloxacin, a new non-zwitterionic fluoroquinolone, can treat gram-positive bacteria like MRSA and many gram-negative pathogens without combination therapy. Delafloxacin is given intravenously or orally. Delafloxacin outperforms levofloxacin against most gram-positive infections, including MRSA and other resistant strains [92].

#### 3.1.2. Fungi

Dermatophyte skin infections can be treated with a variety of antifungal drugs, including griseofulvin, azoles, allylamines, and morpholine derivatives [93]. It is common to utilize polyenes (such as amphotericin B and nystatin) to treat fungal disease because they chemically attach to the fungus’ cell membrane, such as ergosterol, and form cylindrical channels that cause changes in the membrane’s permeability as well as cell death. There are several different formulations of topical antifungal polyenes. The polyene antifungal agents include nystatin, amphotericin B, and pimaricin [94]. Dermatophyte infections should be treated with tablets of griseofulvin. RNA binding and interference with nucleic acid synthesis are all inhibited by griseofulvin, an antifungal obtained from Penicillium griseofulvin. Griseofulvin is effective against dermatophytes. Tinea corporis or cruris in children is treated with griseofulvin, which is given for two to four weeks [94].

Azole antifungal agents have added greatly to the therapeutic options for the treatment of systemic fungal infections by disrupting the fungal 14-demethylase, which has mechanisms to prevent ergosterol synthesis. Ergosterol suppression leads to fungal cell membrane damage, resulting in an increase in cell volume and a decrease in cell division. Azoles can be applied topically or taken orally [95]. Ketoconazole, the first broad spectrum of the azoles group, has confirmed its potential for the severity of fungal infection [96]. The use of medications such as clotrimazole and miconazole has been introduced to minimize the risk of side effects. Sulconazole, tioconazole, and isoconazole, among others, are topical preparations of this type [97]. They are part of the Allylamine family of antifungal agents, including Terbinafine and naftifine. Terbinafine’s ability to combat dermatophytes has been cited as a factor in its success in treating dermatophyte infections [98]. In low concentrations (0.25% cream), amorolfine, a morpholine derivative antifungal, can be effective against dermatophytes, yeasts, and possibly some molds [99]. Skin infections caused by dermatophytes respond well to oral antifungals. When treating dermatophytosis, a daily oral dose of 250 mg of terbinafine is recommended. Tinea pedis, tinea cruris, and tinea corporis of the dry variety show quick and sustained remissions after 2 weeks [100]. A dermatophyte infection caused by *Trichophyton tonsurans*, *Trichophyton violaceum*, or *Microsporum canis* is referred to as tinea capitis [101]. To treat tinea capitis, griseofulvin and ketoconazole are both effective options. *Trichophyton* sp. infections respond well to itraconazole and terbinafine, according to research. Furthermore, griseofulvin can be administered for tinea barbae infections caused by *T. verrucosum* and *T. mentagrophytes*. Itraconazole, fluconazole, and terbinafine are sensitive to these species, while fluconazole is given as a daily treatment of 50 mg for 2–4 weeks in dermatophyte infections of the skin [94,100,102]. Dermatophytes, including *tinea faciei*, *tinea cruris*, and *tinea corporis*, can cause glabrous skin infections. Ketoconazole, itraconazole, fluconazole, and terbinafine are utilized for severe-to-mild instances, according to current studies [103]. Topical azoles such as cream, solution, and spray formulations containing 1% azole as antifungals are the most common. Most require twice-daily administration for 2 to 4 weeks, however some, like bifonazole, are approved for once-daily dosing [95]. Itraconazole, a triazole antifungal agent, has a broad spectrum of activity against infections caused by dermatophytes, yeasts, and many other fungi [104]. Systemic therapy (itraconazole, terbinafine, and fluconazole) can be considered for acute infection caused by the invasion of both *T. interdigitale* and *E. floccosum* [105]. Pityriasis versicolor is caused by *Malassezia* sp., which are found in the human flora and are identified as etiological agents. Ketoconazole and itraconazole are effective treatments for *Malassezia* sp. *Malassezia hyphae* growth was significantly slowed by these therapies, which also caused necrosis in the organisms they were administered to [93,106].

#### 3.1.3. Viral

When a virus infects the body, it causes an illness that can spread to nearly any type of tissue or organ. The main antiviral agents that are commonly used by dermatologists include aciclovir, valaciclovir, famciclovir, vidarabine, ganciclovir, foscarnet, cidofovir, idoxuridine, and zidovudine (De Clercq, 2001 [107]; Park and Han, 2002 [108]).

Herpes simplex is one example of a disease caused by a virus known as herpes simplex virus (HSV), which dwells in nerve endings and is activated during an outbreak and causes pain, sores, and itching [109]. Topical acyclovir, a strong inhibitor of herpes virus DNA, is the recommended treatment for HSv-1 outbreaks. The *varicella zoster virus*, which causes chickenpox, causes herpes zoster, a skin and neurological condition. Within 72 h of the rash emerging, those who are immunocompromised and have moderate to severe pain should begin treatment with oral acyclovir [108,109].

### 3.2. Mucosal Infection

#### 3.2.1. Bacterial

Abnormal vaginal discharge can be caused by an excess of bacteria such as *Gardnerella vaginalis*, *Prevotella* spp., *Peptostreptocci* and *Mobiluncus* spp., as well as other bacteria. Bacterial vaginosis can be treated with metronidazole, tinidazole, and clindamycin, all of which have been approved by the FDA [110]. With the same efficacy, both metronidazole and clindamycin can be applied to the vagina or taken orally. It is more effective against *Gardnerella vaginalis* and *Atopobium vaginale* than clindamycin or metronidazole, but not all *A. vaginae* isolates are metronidazole-resistant [111].

There are fewer cases of bacterial conjunctivitis than viral conjunctivitis. *Haemophilus influenza*, *Streptococcus pneumoniae*, and *Staphylococcus aureus* are the three most prevalent bacteria that contribute to conjunctiva infection [112]. Using a broad-spectrum topical antibiotic is advised. The therapeutic guidelines recommend chloramphenicol 0.5% eye drops and framycetin 0.5% eye drops as initial antibiotics. Eye ointment with 1% chloramphenicol can be applied at night. Conjunctivitis, which causes red eyes, is the most self-limiting infection [35]. Chloramphenicol 0.5% eye drops and 1% eye ointment is recommended as first-line therapy. Fusidic acid 1% eyedrops were recommended as a second therapy until there appears freedom of symptoms for 48 h after application (the usual duration of treatment is 5 days). Fusidic acid has a lower antibacterial activity against Gram-negative bacteria when compared to chloramphenicol. For conjunctivitis caused by *Neisseria gonorrhoeae,* ceftriaxone is the recommended antibiotic [113]. Patients with symptoms of conjunctival hyperemia, mucopurulent discharge, and lymphoid follicle formation should be given oral antibiotics such as azithromycin or doxycycline, which are effective treatments [114].

Vaginal culture for the diagnosis of infection showed that vaginal infections caused by *Chlamydia trachomatis*, *Neisseria gonorrhoeae*, and *Trichomonas vaginalis* were obtained from all subjects in the control cases. Penicillins, cephalosporins, and quinolones are the treatment of choice to treat vaginal infections based on their efficacy and tolerability [115].

#### 3.2.2. Fungi

Oral candidiasis was a more prevalent infection caused by *Candida albicans* that accounts for 50% of cases [116]. Treating this infection requires the use of polyenes including nystatin and amphotericin B in a topical preparation for oral candidiasis therapy. Because polyenes are poorly absorbed in the intestine, the most effective way to treat oral infections is with topical treatment in the form of lozenges and oral suspensions. The group of azoles (fluconazole, miconazole, ketoconazole, and clotrimazole) can also be used to inhibit ergosterol biosynthesis via systemic or oral administration [117].

Candida infection is one of the most common causes of vaginitis that accounts for 85% to 90% of cases [118]. Local azoles are the most common treatment for vaginal *Candida albicans* infection. Clotrimazole, butoconazole, and miconazole should be used for a maximum of three days in combination with local azoles. When it comes to treating a moderate Candida infection, topical azoles outperform local nystatin [119]. The use of fluconazole 150 mg is an alternate treatment for vaginal Candida infection [120].

### 3.3. Pulmonary Infection

There are numerous microbes that can cause pulmonary infections, including bacteria, viruses, and fungi.

#### 3.3.1. Bacterial

As the most prevalent bacteria causing CAP (community-acquired pneumonia), *Streptococcus pneumoniae*, *Haemophilus influenzae*, *Mycoplasma pneumoniae*, *Staphylococcus aureus*, *Legionella species*, *Chlamydia pneumoniae*, and *Moraxella catarrhalis* are found originally in the mouth and nose of infected patients [121]. Hospital-acquired pneumonia (HAP) is the most common cause of resistant bacteria such as *Staphylococcus aureus*, Klebsiella pneumoniae, Pseudomonas aeruginosa, and Escherichia coli [53]. Bacterial pneumonia is treated for 5–14 days, depending on severity, age, comorbidities, initial response, and hospitalization/ICU status. Monitor intravenous therapy length and know when to convert to oral therapy. After clinical improvement and no hemodynamic instability, a patient might be shifted to oral therapy to finish treatment [122]. Levofloxacin, moxifloxacin, erythromycin, and azithromycin are commonly used to treat *M. pneumoniae*. In vitro evaluation using macrolides, especially azithromycin, are most effective against *M. pneumoniae*. Azithromycin treatment could last within 5 days, while tetracycline or fluoroquinolone need 7–14 days [123].

Azithromycin, clarithromycin, erythromycin, or doxycycline are commonly prescribed to patients under the age of 65 who have no risk factors. Patients over 65 years of age with or without comorbidities (chronic heart, lung, liver, or kidney disease, diabetes mellitus, alcoholism, malignancy, and asplenia) are treated with a respiratory fluoroquinolone group (levofloxacin, moxifloxacin, and gemifloxacin), β-lactam combined with macrolides, cefuroxime as an alternative to β-lactam, or macrolide as an alternative [124]. All ages of inpatient ICU and non-ICU including drug resistance are described in Table 1.

Chronic bacterial colonization of the lower airways contributes to airway inflammation, which accelerates the progressive decline in lung function seen in COPD (chronic obstructive pulmonary disease). The course of COPD is characterized by intermittent exacerbations of the disease and shows that approximately half of exacerbations are caused by bacteria [125]. In moderate cases of COPD, antibiotics such as amoxicillin or tetracyclines can be used to treat bacterial infections caused by the disease, co-amoxiclav in moderate/severe cases, and ciprofloxacin in cases in which *Pseudomonas aeruginosa* is a risk factor. In nations with limited pneumococcal macrolide resistance, newer macrolides such as azithromycin, roxithromycin, or clarithromycin are good alternatives in the case of hypersensitivity. Exacerbations of bronchiectasis without the evolving of *Pseudomonas aeruginosa* should be treated with amoxicillin-clavulanate, moxifloxacin, or levofloxacin. Moreover, to treat bronchiectasis caused by *Pseudomonas aeruginosa*, ciprofloxacin or ciprofloxacin plus, anti-pseudomonal B-lactam, or aminoglycoside is commonly prescribed [126].

Tuberculosis (TB) is a lung infection that is mainly caused by the bacteria *Mycobacterium tuberculosis* [127]. The recommended regimens for treating tuberculosis infection are rifampicin, pyrazinamide, and ethambutol for the intense phase and isoniazid and rifampicin for the continuation phase. Furthermore, for TB caused by organisms that are not known or suspected to be drug-resistant, the optimum treatment is a 2-month intensive phase of isoniazid, rifampicin, pyrazinamide, and ethambutol, followed by a 4-month continuation phase of isoniazid and rifampicin [128].

Cystic fibrosis (CF) is an inflammatory disease of the lungs that results in the formation of thick mucus. It is caused by *Pseudomonas aeruginosa*, a bacterium commonly found in the environment. As a conventional treatment, aminoglycosides and β-lactam should be used to treat *Pseudomonas aeruginosa* pneumonia with a de-escalation strategy [53]. Azithromycin’s anti-inflammatory and antivirulence qualities help CF patients’ lung function very well. The Sub-inhibitory azithromycin doses can change *Pseudomonas aeruginosa* bacterial motility, quorum sensing, and virulence expression factors, including protease activity, which may contribute to its pathogenicity [129]. Antimicrobials classified as a class of β-lactams commonly known as ticarcillin, piperacillin, ceftazidime, monobactams (aztreonam), and carbapenem (imipenem, doripenem, and biapenem) are all commonly used to treat *Pseudomonas aeruginosa* infections in cystic fibrosis patients [130]. Inhaled antibiotics such as tobramycin and aztreonam lysate have been documented for cystic fibrosis.

The development and widespread use of nebulized antibacterial therapies such as tobramycin inhalation solution (TIS) have resulted in improved lung function and quality of life. However, the use of nebulizers comes with a number of drawbacks, including lengthy administration times and the need for frequent device cleaning and disinfection. Multiple therapies are required for CF patients, which places a significant burden on patients, and adherence to the recommended treatments remains difficult. Tobramycin inhalation powder (TIP) has been shown to have comparable clinical efficacy and safety to TIS, with improved patient convenience, satisfaction, and treatment adherence. TIP was well tolerated in patients with cystic fibrosis, according to long-term safety studies [131]. Recently, a second-generation tobramycin formulation in an inhalation solution and a dry-powder inhaler (DPI) was also approved in some countries [132,133].

When combined with other diseases such as tuberculosis, COPD, and sarcoidosis, chronic pulmonary aspergillosis (CPA) increases the risk of both morbidity and mortality [134]. First-line medications include itraconazole and voriconazole, which are the only oral treatments having anti-Aspergillus efficacy. The recommendation recommends triazoles such as itraconazole and voriconazole as first-line treatments [135,136].

#### 3.3.2. Viral

The first adenoviruses (Ads) were discovered in human adenoid cells in 1953. They are naked, double-stranded DNA viruses. Based on shared DNA sequences and other biological features, 51 human Ad serotypes have been categorized into six categories (A–F). Although Ads can infect multiple organ systems, some strains prefer to replicate in the respiratory system, where they can cause a broad variety of symptoms, including the common cold, strep throat, tonsillitis, bronchitis, and pneumonia. Therapeutic options for treating illnesses caused by Ads are currently unavailable. There are no specific treatments for severe Ad infections in immunocompromised hosts, but some wide-spectrum antivirals have been used (Abed and Boivin, 2006 [137]).

A. Ribavirin

Although most reports are anecdotal or used a small series of cases, IV ribavirin has shown some apparent success in treating Ad disease in children, such as stem cell transplant (SCT) recipients with pneumonia [138], bone marrow transplant (BMT) recipients with gastroenteritis [139], and leukemic children with disseminated disease [140]. Mild, reversible anemia is the most frequently reported IV ribavirin side effect.

B. Cidofovir

Treatment of symptomatic Ad-associated disease in immunocompromised children and adults with the wide spectrum cytosine nucleotide analogue cidofovir has shown some efficacy [141,142]. Two out of three cidofovir-treated SCT patients recovered, while only three out of thirteen ribavirin-treated SCT patients did [142]. However, nephrotoxicity has prevented widespread IV cidofovir use in clinical practice (Abed and Boivin, 2006).

The group of Paramyxoviridae is some of the world’s deadliest and most medically significant respiratory virus outbreaks. There are two subfamilies of the viral genus Paramyxovirus, both of which are single-stranded, negative-sense RNA viruses. Human parainfluenza virus (HPIV), human metapneumovirus (HMPV), and human respiratory syncytial virus (HRSV) are all examples of important viruses that can cause infections in the upper and lower respiratory tracts of people. Human rhinovirus illness treatment also used ribavirin in the form of nucleoside analogue ribavirin (1-d-ribofuranosyl-1,2,4-triazole-3-carboxamide; Virazole) and was approved for use in the treatment of severe HRSV disease in newborns at high risk [143]. This medicine is effective against both DNA and RNA viruses [144]. There is a use of fusion inhibitors with two parts between the F protein of HRSV (F1 and F2). There are two highly conserved heptad repeat sections (HR1 and HR2) in the F1 subunit. The fusion of viral and cellular membranes is caused by a conformational shift in the F protein that results in a stable HR1/HR2 six-helix bundle [145]. Single-stranded antisense oligonucleotides (ODNs) consisting of 18–25 nucleotides are used to silence mRNA by annealing to their corresponding sequences. Endogenous ribonuclease H (RNase H) is attracted to the freshly formed RNA-DNA hybrid and cleaves the RNA portion of the hybrid [146]. Immunosuppressive drugs used for the avoidance of HRSV illness have been approved. It has been suggested that high-risk newborns receive an intravenous dose of RespiGam, a polyclonal immune globulin enriched in neutralizing antibodies against HRSV. In patients with HRSV, RespiGam resulted in a 41% decrease in hospitalizations, a 53% decrease in hospital stays, and a 44% decrease in overall HRSV-related intensive care unit days [147]. Glucocorticoid therapy has been proposed as a means of reducing the pulmonary inflammatory reaction that occurs in the wake of HRSV infection [148].

Coronaviruses were first found in the human embryonic trachea and nasal epithelium organ cultures and basic human kidney cell cultures 40 years ago [149]. The single-stranded, positive-sense RNA genome of these viruses is about 30 kb, which is thought to be the biggest self-replicating RNA known [150]. The virus that caused the SARS outbreak in 2002 and 2003 was found to be a new coronavirus (SARS-CoV). This virus caused more than 8000 cases of SARS and 800 deaths in 26 countries [151,152].

At the time of the SARS outbreak, there was no clear way to stop the disease from getting worse. But the study in that area has been stepped up, and this has led to some promising treatments that still need to be tested during future outbreaks (Abed and Boivin, 2006).

- Inhibitors of proteases

HIV protease inhibitors such as lopinavir and ritonavir could potentially be used to treat SARS because they have been shown to kill the SARS-CoV virus in a lab setting [153,154]. In a small group of people who got lopinavir/ritonavir as their first treatment, the rates of intubation and death went down by a lot [153]. Chu et al. (2004) also looked at how the treatment of lopinavir/ritonavir compared to historical controls. All of the patients were also given ribavirin and steroids. In the lopinavir/ritonavir group, the chances of getting ARDS or dying within 21 days were much lower than in the historical controls. Nosocomial infections also went down in the group that did something. Because they work against SARS-CoV in test tubes, other protease inhibitors could be used to treat SARS. These are nelfinavir, calpain inhibitor III (Z-Val-Phe-Ala-CHO), and calpain inhibitor IV (Val-Leu-CHO) [155].

b. siRNA

RNA interference treatment is a method in which small interfering RNAs (siRNAs) cause mRNAs with the same sequence to break down. In vitro, specific siRNAs that target the replicase 1A region of SARS-CoV showed a strong effect on stopping the replication of different types of SARS-CoV [156]. However, research into their medicinal potential is ongoing (Abed and Boivin, 2006).

### 3.4. CNS Infection

#### 3.4.1. Bacterial

Bacteria and viruses that attack the brain, cerebellum, spinal cord, and optic nerves could cause a wide range of serious health complications [157]. Meningitis, encephalitis, and abscesses are three types of acute central nervous system (CNS) diseases. For example, *Streptococcus pneumoniae*, *Neisseria meningitidis*, *Group B Streptococcus Suppuratives*, *Listeria monocytogens*, and *Haemophilus influenzae* can all cause bacterial meningitis [158]. Drug penetration of the blood–brain barrier should be taken into account while designing the treatment, and this includes lipid solubility, molecular size, and molecular structure. Third-generation cephalosporins, such as ceftriaxone and cefotaxime, have higher penetration than previous generations. Meningitis medications from other classes, such as macrolides, tetracyclines, and quinolones, do not penetrate the blood–brain barrier well enough to be utilized as a first-line therapy [159].

Empirical treatment for preterm to <1-month patients with suspected meningitis but with Gram-negative stains or cultures is ampicillin IV plus cefotaxime IV. For patients aged 1 month to 50 years experiencing infection caused by the presence of *Streptococcus pneumonia*, *Neisseria meningitides*, *Haemophilus influenzae*, and *Listeria* sp., the use of treatment with ceftriaxone IV or cefotaxime IV plus vancomycin is recommended. For patients that are 50 years old, treatment is preferred with ampicillin IV plus ceftriaxone IV or cefotaxime IV plus vancomycin IV [160,161]. Therapy for bacterial meningitis (suggested by Gram stain or culture) by specific microorganisms is described in Table 2.

The clinical manifestation of a brain abscess can include headache, fever, cranial-nerve palsy, or gait disorder that may occur following head trauma or neurosurgical procedures. Most patients with brain abscesses may develop a localized bacterial infection in the parenchyma along with inflammation and in some cases edema [162]. In pneumococcal infection models, the use of clindamycin to treat both sepsis and meningitis markedly improves CNS pathology in part because clindamycin appears to induce programmed cell death pathways leading to filamentation rather than lysis [163]. A combination of high-dose penicillin (10–20 million units per day) and metronidazole (15 mg/kg IV every 12 h) should be tried in this case. Brain abscesses caused by chronic otitis media and mastoiditis are best treated with a combination of cefotaxime, ceftazidime, or ceftriaxone plus metronidazole, among other antimicrobials. *Enterobacteriaceae* and *Pseudomonas* spp. are the microorganisms addressed by this regiment [161,164]. The antibiotic treatment can be modified using third-generation cephalosporins ceftazidime IV every 4 h and ceftriaxone IV every 12 h. The regiment is needed when antimicrobial susceptibility testing results are available. Vancomycin plus cefepime and metronidazole is an empirical treatment for patients who have neurosurgical procedures or head trauma. Vancomycin should also be considered for patients who are suspected to have staphylococcal infection [165]. In conjunction with a third-generation cephalosporin, treatment with rifampicin or daptomycin has been proven to be beneficial in eradicating pneumococci and reducing the production of a pro-inflammatory molecule [166].

#### 3.4.2. Viral

Exogenous enteroviruses, arboviruses, HSV, varicella-zoster virus (VZV), mumps virus, CMV, Epstein–Barr virus (EBV), adenoviral infections, and the human immunodeficiency virus (HIV) cause the vast majority of viral meningitis cases (HIV). Enterovirus and arbovirus infections do not have a specific treatment. Encephalitis caused by enteroviruses has modest symptoms and can usually be resolved within 7–10 days without the use of antiviral drugs. HSV and VZV meningitis patients can be treated intravenously with acyclovir [161].

#### 3.4.3. Fungi

Fungal infections are particularly life-threatening for immunocompromised hosts because they can spread rapidly and invade the central nervous system. People with haematological malignancies, organ transplant patients, and HIV-positive people are all especially at risk. Yeasts, Aspergillus species, and an expanding list of molds and dimorphic fungi, many of which are endemic to specific regions, are all possible pathogens [167]. Polyenes such as amphotericin B and its lipid formulations, azoles such as fluconazole and itraconazole, and the newer voriconazole and osaconazole are all effective antifungal therapies [168]. The central nervous system is usually impermeable to members of the new antifungal class of echinocandins, which includes caspofungin, micafungin, and anidulafungin. At the beginning of therapy for Candida and Cryptococcus neoformans CNS yeast infections, amphotericin B and flucytosine are used [169]. For *Aspergillus* sp., voriconazole is recommended, but amphotericin B, especially in the lipid version, can be effective as well [170]. Posaconazole shows promise for the zygomycetes and possibly some of the rarer pigmented fungi, but amphotericin B preparations are still suggested due to a lack of reliable treatment data for CNS infections with most of the non-Aspergillus molds [171]. CNS manifestations of Coccidioides can be treated with oral fluconazole, while histoplasmosis and blastomycoses are usually treated with amphotericin B. The group at risk of central nervous system (CNS) fungal infections can be difficult to accurately diagnose, making effective treatment difficult to achieve [167,172].

### 3.5. Gastrointestinal Infection

The gastrointestinal (GI) tract is a tract that consists of the mouth to the anus and related organs, including the liver, pancreas, and gallbladder [173]. Infections in the digestive tract are caused by various kinds of bacteria, protozoa, and viral pathogens [174]. Diarrhea, nausea, vomiting, and abdominal pain are all signs of gastroenteritis. Diarrhea and severe or life-threatening dehydration can be caused by a variety of enteric infections, including viruses (rotavirus and norovirus) and bacteria (*Escherichia coli*, *Clostridium difficile*, *Campylobacter jejuni*, and *Vibrio cholerae*) [175]. Dehydration is the main danger of these infections, so the first part of treating these infections is to prevent and treat dehydration [176,177]. The following are conventional treatments used in gastrointestinal infections based on the cause of the infection.

#### 3.5.1. Bacterial

Several different types of bacteria can be found in the digestive tract, leading to a variety of infectious diseases through the production of virulence factors, such as toxins, that interact with the intestinal mucosa. A contaminated food source could also lead to the growth of bacteria and the production of toxins. Heating can kill microorganisms in food, but the toxin is unaffected and can have an effect within hours of consumption [176,178]. Infection with *Helicobacter pylori* is a significant risk factor for developing gastric and duodenal ulcers, as well as gastric adenocarcinoma and gastric mucosa-associated lymphoid tissue (MALT) lymphoma [176]. Therefore, treatments that can be done to reduce the severity in case of bacterial infection were presented in Table 3.

#### 3.5.2. Viral

In some situations, gastrointestinal infections were caused by viruses and bacteria, but in a minor and self-limiting incident, while in others they can be severe and require special care [179]. Adenovirus serotypes 40 and 41, rotavirus, astrovirus, and caliciviruses (noroviruses, sapoviruses) cause most viral gastroenteritis. Molecular approaches are being used in clinical laboratories to diagnose viral gastroenteritis. In patients with HIV or compromised immunity, viruses like cytomegalovirus and herpes simplex virus can cause enteric disorders, but they are diagnosed by testing gastric or intestinal biopsy tissues rather than stool [179]. Pathogenic viruses have the potential to cause local and systemic complications. Viruses can disrupt the intestinal mucosal barrier by related mechanisms and lead to increased diarrhea as well as tissue invasion [180]. Treatment of infections of the gastrointestinal tract caused by viruses is shown in Table 4.

#### 3.5.3. Protozoa

Protozoa are parasites transmitted through contaminated water and food consumption and mainly affect children and the elderly and cause considerable health problems [181]. Single-celled protozoan parasites can quickly multiply in the body, leading to the development of serious infections. Most protozoal infections tend to be asymptomatic. Protozoal infections can also cause structural and functional abnormalities of the small intestine in humans and can be misdiagnosed as appendicitis or other inflammatory diseases of the gastrointestinal tract [182]. Treatment of infections in the gastrointestinal tract caused by these protozoa can be seen in Table 5.

### 3.6. Lymphatic Infection

#### 3.6.1. Bacterial

Acute streptococcal or staphylococcal skin infection (called cellulitis) or an abscess in the skin or soft tissue commonly causes lymphadenitis and lymphangitis, which are infections of the lymph nodes and lymph channels. Septicemia is a life-threatening illness that can occur if germs enter the bloodstream (blood poisoning) [183]. Treatment of both depends on the infecting organism [184]. In lymphadenitis, antibiotic treatment generally helps to cure the primary bacterial infection. Maintaining overall health is very helpful in preventing infection [185]. It is recommended that *Staphylococcus aureus* and the group A β-hemolytic streptococci be used to treat acute bacterial cervical lymphadenitis. Cloxacillin, cephalexin, or clindamycin are all appropriate oral antibiotics [186]. Ciprofloxacin has shown activity against several atypical mycobacteria and may be considered among the potential drugs to treat lymphadenitis [187].

Lymphangitis occurs due to infection in the sewer that occurs when microorganisms enter the lymphatic tract directly through abrasion or a wound and can also occur as a complication of an infection. The more common cause is species of β-hemolytic streptococci [188]. Lymphangitis can be treated with appropriate antibiotics; the most commonly used antibiotics being penicillin or first-generation cephalosporins. In addition, patient therapy may include second- or third-generation cephalosporins, such as cefuroxime (Ceftin), ceftriaxone (Rocephin), or penicillinase-resistant synthetic penicillin [185,188]. Antibiotics are indicated to treat cellulitis as well as other hidden infections [185].

Penicillin therapy may be sufficient but is used for 1 week instead of the commonly used dicloxacillin or cephalexin to ensure anti-staphylococcal protection. Then, if known to be allergic to penicillin, another choice of medication, such as clindamycin, erythromycin, and levofloxacin, can be used (Ferri, 2016 [189]).

#### 3.6.2. Viral

Antigens in the interstitial fluid are cleaned up by peripheral lymph nodes, which are found deep in the subcutaneous tissue. A healthy lymph node should measure less than one centimeter in circumference. In most cases, an infectious illness, either local or systemic, that is mild and self-limiting causes lymphadenopathy (LAP). On the other hand, it might be a sign of a more serious cancer. More than half of all LAPs occur in the head and neck, making up 75% of all localized LAPs [190].

Acute bilateral cervical lymphadenitis is a possible symptom of generalized lymphadenopathies associated with infection (LAP), such as that produced by the Epstein–Barr virus or cytomegalovirus [190].

Clinicians may encounter difficulties in making a diagnosis of lymphadenopathy due to a wide variety of possible reasons. Getting a complete history and physical, making a list of differential diagnoses, and sorting them by how they appear are crucial for making an accurate diagnosis. Lymphadenopathy can result from a wide variety of factors. Cytomegalovirus, hepatitis, herpes simplex, HIV, mononucleosis, rubella, and viral pharyngitis are all examples of viruses that can cause illness [68,191,192]. Depending on culture and sensitivity results, acute bacterial cervical lymphadenitis without a known primary infectious source should cover *Staphylococcus aureus* and *Streptococcus pyogenes* with the use of oral cloxacillin, cephalexin, cefprozil, or clindamycin. Methicillin-resistant *S. aureus* resists numerous antibiotics. Vancomycin is used for difficult infections, whereas trimethoprim-sulfamethoxazole or clindamycin works well for outpatient treatment [191].

#### 3.6.3. Parasites

On Alor Island in East Nusa Tenggara, Indonesia, lymphatic filariasis caused by Brugia timori and Wuchereria bancrofti is a serious issue for public health [193]. Microfilaria (mf) density and short-term reactions to either a single dose of diethylcarbamazine (DEC) (6 mg/kg body weight) and albendazole (Alb, 400 mg) or a divided dose of DEC (100 mg on day 1 and the rest on day 3) were studied in order to implement a control programme [194]. Thirty people were hospitalized with *B. timori* infections, and the findings were compared to those obtained from the treatment of twenty-seven people with *W. bancrofti* infections in an effort to determine the optimal treatment protocol [193]. Alb can be used to treat *B. timori* infection in addition to DEC, and there were no extra side effects observed when compared to DEC treatment alone. The use of DEC and Alb to control lymphatic filariasis may also have an effect on the control of intestinal helminths in Indonesia, a country with a high prevalence of intestinal helminths [193,195].

## 4. A New Approach of Antimicrobials Drug Delivery Systems for the Treatment of Infectious Diseases through Stimuli-Responsive Development

### 4.1. Skin and Soft Tissue Infection

#### 4.1.1. pH-Responsive Delivery System

**Figure 1:**
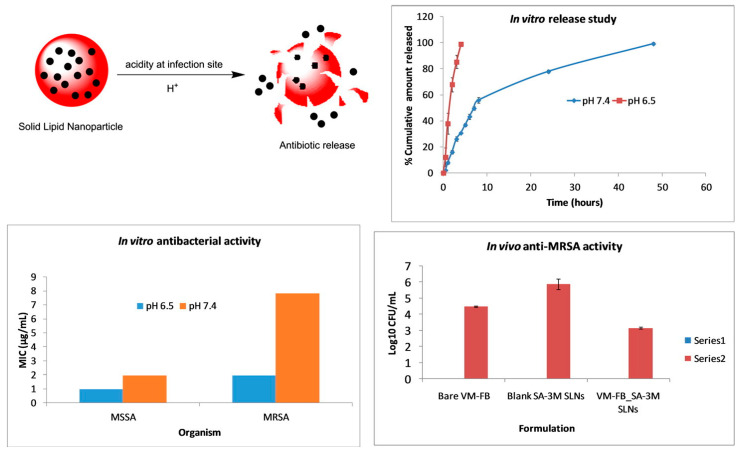
A graphical abstract of greater drug release, followed by enhanced in vitro and in vivo antibacterial activity of solid lipid nanoparticles at an acidic pH. Reprinted with permission from [202]. Copyright 2017 Elsevier Inc.

pH is a typical drug release stimuli among a variety of other stimulation. Infection, cancer, and inflammation are all disorders that can be targeted by manipulating pH levels [196,197]. Various researchers had reported that the pH level at infection sites is usually lower than the physiological value of 7.4 [198,199,200,201]. Therefore, to treat skin infections caused by methicillin-susceptible and resistant *Staphylococcus aureus* (*S. aureus*) (MSSA and MRSA), pH-responsive solid lipid nanoparticles (SLNs) were created using a vancomycin base (VM-FB) and a synthesis product of an acid cleavable lipid (VM-FB SA-3M). The particle size (PS) and zeta potential in the research were found at 132.9 ± 9.1 nm, 0.159 ± 0.01, and −26 ± 4.4 mV, respectively, with 57.80 ± 1.1% encapsulation efficiency, and the release was faster at pH 6.5 than 7.4. Additionally, SLNs were more active at pH 6.5 than 7.4 when evaluated in vitro. More than 22 times less MRSA had also been found in the VM-FB SA-3M SLN treated animals compared to the VM-FB only, as shown in Figure 1 [202].

**Figure 2:**
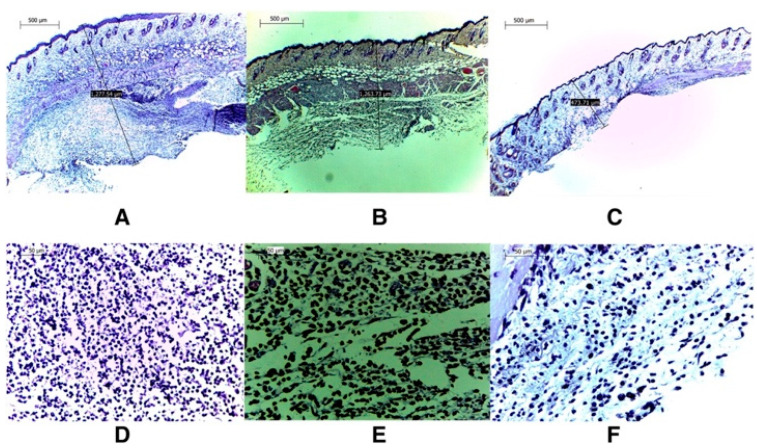
Further histological examinations confirmed VM-FB_SA-3M_SLNs’ efficacy against MRSA infections. Images taken at 4× magnification revealed that the skin thickness of mice treated with blank SA-3M_SLNs was 1277.54 m (**A**), whereas the skin thickness of mice treated with bare VM-FB was 1263.73 m (**B**), and that of mice treated with VM-FB_SA-3M_SLNs was 473.71 m (**C**). (**F**) shows that skin treated with VM-FB_SA-3M_SLNs showed little evidence of inflammation, but skin treated with blank SLNs and bare VM-FB showed a much higher number of lymphocytes and leukocytes (**D**,**E**). Reprinted with permission from [202]. Copyright 2017 Elsevier Inc.

Histological tests further confirmed the efficacy of VM-FB SA-3M SLNs in treating MRSA infections. Specifically, mice treated with blank SA-3M SLNs and VM-FB only showed a skin thickness of 1277.54 µm (Figure 2A) and 1263.73 µm (Figure 2B), respectively. Greater skin thickness in the animals treated with VM-FB_SA-3M_SLNs was found at 473.71 µm (Figure 2C), and it was less inflamed (Figure 2F). An increasing number of lymphocytes and leukocytes were observed in skin treated with blank SLNs and VM-FB only (Figure 2D,E).

**Figure 3:**
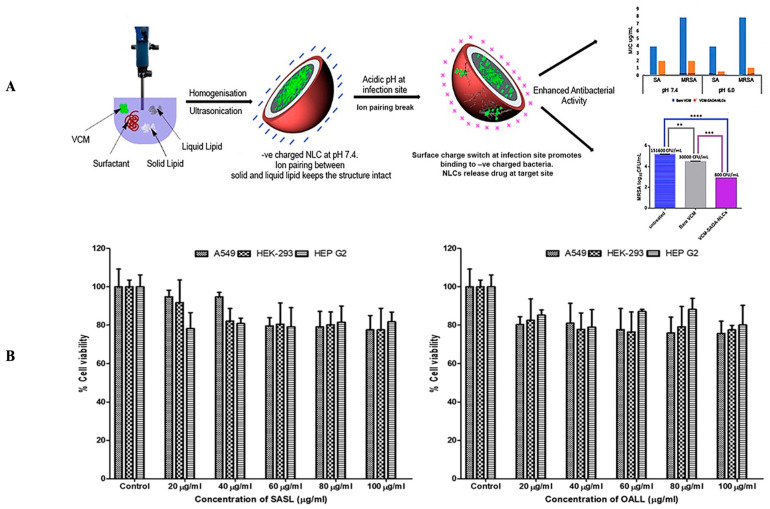
(**A**) A graphical abstract of the preparation of pH-responsive nanostructured lipid carriers for the intravenous delivery of vancomycin against resistant and sensitive *Staphylococcus aureus* bacteria. Reprinted with permission from. [205]. Copyright 2019 Elsevier Inc. (**B**) A cytotoxicity evaluation of various concentrations of stearic acid-derived solid lipids and oleic acid-derived liquid lipids against A 549, HEK-293, and HEP G2 cells. Reprinted with permission from [205]. Copyright 2019 Elsevier Inc. ** *p* < 0.01; *** *p* < 0.001 and **** *p* < 0.0001.

These findings concluded that SA-3M could form pH-responsive SLNs that specifically release antibiotics at acidic infection sites [202]. Another approach of vancomycin (VCM)-loaded SLNs using NMEO (N-(2-morpholinoethyl) oleamide) (VCM_NMEO_SLNs) had 302.8 ± 0.12 nm as the particle size, and the polydispersity index (PDI), zeta potential (ZP), and entrapment efficiency (EE) of the formulation were 302.8 ± 0.12 nm, 0.23 ± 0.03, −6.27 ± 0.017 mV, and 81.18 ± 0.57%, respectively. The investigation of drug release and antibacterial activity had shown much higher levels at pH 6.0 rather than pH 7.4, while in silico investigations have revealed molecular pathways for better stability and drug release. Furthermore, mice treated with VCM_NMEO_SLNs exhibited a 4.14 times greater reduction in their MRSA burden compared to mice treated with VCM only (*p <* 0.05). According to the findings of the study, pH-responsive SLNs can be made using NMEO and may be useful in the treatment of bacterial infections [203]. In addition to that, the formulation of pH-responsive liposomes for the targeted distribution of VCM uses two-chain fatty acid-based lipids (FALs) and has been conducted with amino acid head groups known as Di-Oleoyl Amino Propionic Acid (DOAPA-VCM-Lipo) and Di Linoleoyl Amino Propionic Acid (DLAPA-VCM-Lipo). The in vitro evaluation of *S. aureus* and MRSA bacteria had shown that both pH 7.4 and 6.0 had greater antibacterial activity after 72 h. When examined using flow cytometry, these compounds killed MRSA cells at a high rate (71.98% for DOAPA-VCM-Lipo and 73.32% for DLAPA-VCM-Lipo). Therefore, this study’s pH-responsive two-chain (FAL) liposomes could help with VCM-targeted delivery [204]. For the intravenous delivery of VCM against resistant and sensitive *S. aureus* bacteria, this study used novel fatty acid-derived lipids (stearic and oleic acid) to develop a new approach to pH-sensitive VCM-loaded nanostructured lipid carriers (NLCs) integrating solid and liquid fats (Figure 3A). The PS was 225.2 ± 9.1 nm, and the antibacterial activity had shown that VCM-loaded NLCs outperformed bare VCM against methicillin-susceptible and resistant *S. aureus*. In a cell viability study, VCM-loaded NLCs killed 2.5 times more cells than the drug alone at similar concentrations (Figure 3B). When tested in an MRSA skin infection mouse model, the efficacy showed that dermal VCM-loaded NLCs reduced the MRSA CFU load by 37-fold compared to bare VCM (*p <* 0.05). These novel pH-responsive NLCs may, thus, improve antibiotic delivery efficiency [205].

Ciprofloxacin (CIP) and 3-amino-7-chloro-2-nonylquinazolin-4(3H)-one (ACNQ), a quorum sensing inhibitor (QSI), could now be delivered to mature *Pseudomonas aeruginosa* biofilms in a new way. That is, charge–charge interactions between alginate NPs and CIP were used to create alginate nanoparticles (NPs) capable of encapsulating CIP. When CIP and QSI were released simultaneously from pH-responsive NPs in a biofilm model, the biofilm’s vitality was reduced, and it considerably reduced bacterial burden (CIP-only *p*-value = 0.0001, ALG_ALD_CIP *p*-value = 0.0001, and ALG_QSI_ *p*-value = 0.0048). However, when the QSI and CIP were combined in the alginate nanoparticle formulation, they totally eradicated the infection (*p*-value 0.0015). The QSI drugs released more cumulatively from ALGQSI at biofilm-relevant pH 6.0 (0.5 M citrate buffer) than at pH 7.4 (0.1 M phosphate buffer), which was attributed to increased hydrazone bond cleavage at the lower pH. Furthermore, alginate NPs had deeply pierced the bacterial biofilms, probably due to their charges and the release of the QSI agent. Combination of medicines that prevent the production of biofilms while simultaneously eliminating mature *Pseudomonas aeruginosa* biofilms could be extremely beneficial for the pH-responsive delivery system [206].

#### 4.1.2. Enzyme-Responsive Delivery System

Enzymes were used as stimuli in the design of smart DDSs in recent years due to their unique superiorities, such as substrate specificity and high selectivity under mild conditions [207,208,209,210]. By emitting a visual signal, bacteria enzymes’ breakdown of nanocarriers can be stopped at the source. Octenidine had also been used to study the effects on primary human dermal microvascular endothelial cells, which play an important role in cutaneous wound healing, using fluorescent-labeled hyaluronan nanocapsules containing polyhexanide biguanide and poly-L-lactic acid NPs. Notably, endothelial cell adhesion molecules and cytokines are not affected by enzyme immunoassays. Moreover, when dermal fibroblasts were co-cultured with angiogenic stimulators, their ability to generate capillary-like structures was unaffected. Antimicrobial nanocomposites could, therefore, be a safe and effective method of preventing wound infection, according to the study’s findings [211]. Further research revealed that MRSA’s intra-phagocytic survival ability protects them from antibiotics’ bactericidal action, causing recurring of infections [212]. It is, therefore, critical to develop delivery systems that could target macrophages and kill intracellular MRSA. Monocytes or macrophages, alveolar macrophages, astrocytes in the brain, and hepatocytes in the liver all have mannose receptors (MRs) [213,214].

**Figure 4:**
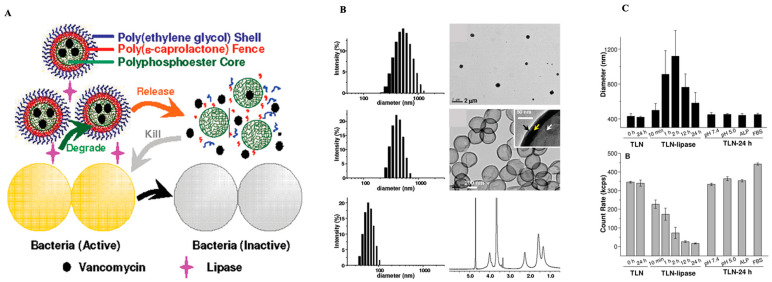
(**A**) Polymeric triple-layered nanogel with a poly(-caprolactone) interlayer between the cross-linked polyphosphoester core and the polymer shell, which had been activated by bacterial lipase to treat bacterial infections (ethylene glycol). (**B**) Triple-layered nanogel in THF size distribution and the TEM picture; the size distribution of triple-layered nanogel in water and a TEM picture; and the cross-linked polyphosphoester core, the poly(-caprolactone) molecular fence, and the polyethylene glycol shell. The D2O 1H NMR spectrum of the triple-layered nanogel in D2O; the size distribution of mPEG-PCL micelles; and the 1H NMR spectrum (ppm). The white arrow in the subpictures B indicates the cross-linked polyphosphoester core; the yellow arrow indicates the PCL molecular fence, and the black arrow indicates the shell of PEG (**C**) Culturing in media for varied periods resulted in changes in the diameter (intensity statistics) and the count rate. Reprinted free access from [216]. Copyright 2012. Journal of the American Chemical Society.

Propidium iodide (PI), a cell membrane-impermeable fluorescent dye, was utilized as a model because mannosylated ligands interact with MRs. Further, bacteria release enzymes such as phosphatase and phospholipase, which degrade the polyphosphoester core and accelerate the release of the medication. This was validated by incubating vancomycin (VAN)-loaded nanogels (NG-V) with alkaline phosphatase in a Tris-HCl buffer at pH 7.4 for 24 h. After 72 h in the buffer, the VAN release from NG-V was 24.3 ± 0.1%; however, after 72 h of incubation with 500-unit mL^−1^ of alkaline phosphatase, it had increased to 67.0 ± 1.4%. Furthermore, treatment of NG-V with 10 mM of dithiothreitol increased the cumulative release of VAN due to the disulfide linkages in NG-V [215]. Following this, antimicrobials were delivered to bacterial infection sites using a lipase-sensitive polymeric triple-layered nanogel (TLN). Notably, an amphiphilic diblock copolymer was used to make the TLN. In an aqueous solution, a hydrophobic and compact molecular fence was formed by collapsing hydrophobic poly(-caprolactone) (PCL) segments around the polyphosphoester core (Figure 4A). Once lipase-secreting bacteria were found, the TLN–PCL barrier disintegrated and released the antibiotic. It was discovered that the NPs had a spherical shape and that they had a three-layered structure, one of which was saturated. According to TEM, the average diameter of the TLN was approximately 420 nm, while the average interlayer thickness was approximately 29 nm. The micelles generated in water via the macroinitiator monomethoxy poly(ethylene glycol)-b-poly(ε-caprolactone) (mPEG-PCL) alone, also had an average diameter of 56 nm, which is noteworthy (Figure 4B). That is, only in the presence of *S. aureus* did the TLN release nearly all of the encapsulated VAN within 24 h, greatly limiting the growth of *S. aureus* (Figure 4C) [216].

Following this, the TLN injected intracellular microorganisms. When lipase-secreting bacteria create an infection, there is an effective and universally safe treatment [217]. Since many pathogenic Gram-positive bacteria (e.g., *streptococcus*) produced the enzyme hyaluronidase, which could degrade the hyaluronic acid (HA), a new class of HA-based nanocapsules containing the antimicrobial agent polyhexanide was developed. Furthermore, the interaction between hydrophilic hyaluronic acid and NCO groups of 2,4-toluene diisocyanate (TDI) formed polymeric capsules. The amount of released material from different nanocapsules was not significantly different after incubation, with the lowest concentration of hyaluronidase at 1 mg/mL^1^. Therefore, the antimicrobial agent effectively killed the pathogenic bacteria with MIC values at 125 and 500 μg·mL^−1^ against *S. aureus* and *E. coli*, respectively. These results indicate that the nanocapsules are bactericidal against both Gram-positive and Gram-negative bacteria [218]. In the process of damaging bacterial cells, when enzymes such as penicillin G amidase (PGA) and β-lactamase (Bla) were activated, antibiotic-loaded polymeric vesicles self-immolate structurally and morphologically, resulting in a prolonged antibiotic release. Topical treatment of MRSA-infected burn wounds with VAN-loaded PC1 vesicles was performed as a proof-of-concept. In the in vivo murine-infected burn wound model, treatment with VAN and VAN-loaded PC1 vesicles improved wound healing compared to the control groups. In summary, PGA and Bla-responsive polymeric vesicles were created, and enzyme-mediated degradation and microstructural evolution were investigated. The use of antibiotic-loaded responsive vesicles resulted in improved structural stability, reduced side effects, the feasibility of combination therapy, bacterial strain-selective delivery of powerful antibiotics, and improved burn site healing. Overall, the modular design of enzyme-responsive vesicles from amphiphilic block copolymers, which share the same backbone scaffold but are connected to diverse types of enzymatic substrates in the side chain, was successful. The drug stability, adverse effect reduction, and strain-specific drug release had also all been enhanced. Additionally, an in vivo mouse model with improved wound healing and antibiotic release from polymeric vesicles that are degradable via Bla were used to demonstrate the efficacy of the treatment. As a result of high local density and a hydrophobic environment, enzyme-produced primary amines undergo inter- and intramolecular amidation. That is, the crosslinking of NPs is caused by an intermolecular amidation reaction. Enzymes can directly break down aggregates and thereby crosslink them. Notably, this crosslinking chemistry is similar to cross-linked polymersomes generated by light irradiation [217].

#### 4.1.3. Redox-Responsive Delivery System

Hydrogels containing ferrocene (Fc) and redox-responsive ferrocene (Fr) were becoming increasingly popular in the fields of material science, medicine, and catalysis. An aldehyde, Fc, and a dentritic triethylene glycol side chain tetrablock terpolymer based on polynorbornene were produced using a third-generation Grubbs catalyst. Fc groups and triazole groups make it possible to decrease and stabilize the silver NPs (AgNPs). The Schiff’s base reaction between aldehyde groups and side-chain amino groups could also be employed to crosslink gelatin hydrogels using this copolymer. Gelatin hydrogels containing Fc were reduced in situ using AgI to Ag0 by mixing this copolymer with gelatin and AgNO_3_ or by immersing the gels in the AgNO_3_ solution. Notably, antibacterial activity against *E. coli* and *S. aureus* was confirmed during inhibition zone testing using the composites [219]. Moreover, RBC-nanogels with a combination of antiviral and antibiotic properties for MRSA infection were described. Therefore, membrane vesicles templated in in situ gelation were used to coat the RBC membrane onto the nanogel using cystine dimethacrylate (CDA) as the disulfide bond-based crosslinker and L-cystine as the reactive crosslinker. In the RBC-nanogels, VAN had also been present. In the extracellular environment, RBC-nanogels successfully neutralized MRSA-associated toxins, facilitating macrophage absorption. Faster drug release from RBC-nanogels in an intracellular-reducing environment also led to greater bacterial inhibition. Additionally, with intracellular MRSA bacteria, the RBC-nanogels greatly reduced bacterial growth in macrophage cells. Therefore, a novel antibacterial agent for MRSA may be found in the RBC-nanogel system, as demonstrated by these research findings [220].

#### 4.1.4. Temperature-Responsive Delivery System

Targeted, specific antimicrobial delivery requires new, efficient, and transferable methods. Low-temperature-sensitive liposomes and magnetic resonance-guided high-intensity focused ultrasound heating are used to deliver antibiotics. A low temperature-sensitive liposome (LTSL) containing an antimicrobial agent (ciprofloxacin) for induced release at mild hyperthermia (~42 °C), followed by in vitro characterization of release and efficacy against *Staphylococcus aureus* plankton and biofilms, is used to determine the feasibility of localized ciprofloxacin delivery in combination with MR-HIFU hyperthermia in a rat model. LTSLs filled actively with ciprofloxacin were tested using disc diffusion, MBEC biofilm device, and scanning electron microscopy (SEM). Fluorescence spectroscopy and MR-HIFU were used to measure LTSL ciprofloxacin release in physiological buffers and rats. The LTSL emitted >95% ciprofloxacin at 42 °C, but only 5% at 37 °C. Precise hyperthermia exposures in the thigh of rats using MR-HIFU during intravenous (i.v.) LTSL delivery resulted in a fourfold higher local concentration of ciprofloxacin compared to controls (free ciprofloxacin + MR-HIFU or LTSL alone). Ciprofloxacin biodistribution in cold tissues was comparable between groups. Compared to free ciprofloxacin or LTSL at 37 °C, triggered release at 42 °C killed more *S. aureus* bacteria, deformed membranes, and altered biofilm matrix. This method could give high-concentration antimicrobials to chronic wounds.

#### 4.1.5. Magnetic-Responsive Delivery System

Utilizing superparamagnetic iron oxide nanoparticles (SPION) allowed the efficacy of this method in removing biofilms. The magnetothermally successfully stimulated the release of antibiotics from drug-delivery carriers. Iron oxide polymersomes (IOPs), which were formed from methoxy poly(ethylene glycol)-b-poly(d,l-lactic acid) diblock co-polymers to co-encapsulate methicillin within the aqueous core and hydrophobic SPIONs within the membrane bilayer, were used. Controlled release of a model antibiotic, ofloxacin, was achieved by embedding Ag NPs in a poly(butyl methacrylate-co-acrylamide-co-methacrylic acid) hydrogel, and then irradiating the mixture with a 405 nm laser for 15 s at 10, 30, and 50 min after the experiment began. Ofloxacin was released from these samples at a considerably higher rate (>80% of total mass) after 70 min than from the non-irradiated samples [221].

#### 4.1.6. Light-Responsive Delivery System

Several wavelengths of light have been studied as potential initiators in the fight against bacterial diseases [222,223]. These include ultraviolet (UV) light, visible light, and near-infrared (NIR) light. One of the successful strategies for infection control is the use of light to trigger the release of drugs from nanocarriers [224,225,226]. With the help of a photosensitive nanogel, Ballesteros et al., 2020 were able to immobilize AgNPs on the surface of poly(-caprolactone) nanofiber carpets. The nanogel disintegrated when exposed to UV light (405 nm), triggering the controlled release of AgNPs into nanofiber mats and the following release of Ag+, both of which are effective at killing bacteria [224]. Nitric oxide (NO) and carbon monoxide (CO) have both been reported to be released from nanocarriers in response to UV light and visible light, respectively, to treat bacterial infections of incisions [225,226]. However, visible and UV light’s inability to reach deeper layers of the skin may restrict its usefulness [227].

In 2019, Wang et al. used PVA hydrogel infused with Ag-doped TiO_2_ (Ag/TiO_2_) nanoparticles to heal an infected wound. Doping the surfaces of TiO_2_ NPs with Ag significantly increased their photodynamic activities in the visible frequency range. Furthermore, TiO_2_ NPs suppressed the possible toxicity of Ag+ by inhibiting Ag+ release. Within 5 min of being exposed to 606 nm visible irradiation, the hybrid hydrogel displayed potent bactericidal abilities against *E. coli* and *S. aureus* via a light-induced generation of reactive oxygen species (ROS) in the Ag/TiO_2_ system. When applied to a wound, this hybrid hydrogel substantially reduced *S. aureus*-induced infection and hastened the healing process [228].

### 4.2. Mucosal Layer Infection

#### 4.2.1. Vaginal Infection

An ideal host–microbiota relationship in the vagina governs female reproductive health. Bacterial vaginitis (BV) causes 50% of vaginitis cases and is caused by an imbalance in the types of bacteria normally found in the vagina. Therefore, the drug delivery system should stay at the infection site for a long time to be effective. Unlike other systems, the vagina is highly dynamic in drug absorption, metabolism, and elimination [229]. Therefore, antimicrobial medications should be concentrated at the infected sites because of the increasing prevalence of vaginal infections, particularly among women of reproductive age. Direct therapeutic action, lower drug doses and adverse effects, and self-insertion are all advantages of topically applied therapy. However, variations in vaginal physiological parameters significantly affect vaginal medication delivery efficacy. Moreover, due to their limited retention time and discomfort, patients are less likely to adhere to conventional vaginal dosing forms. Scientists have, therefore, recently created nano-systems, mucoadhesive systems, and stimuli-responsive systems to circumvent these constraints [230].

##### pH-Responsive Delivery System

Vaginal pH is a crucial variable in VDDS because it has a major impact on how well a product works. Infection and the existence of seminal fluids both contribute to an increase in pH. Hydrogel isn’t the only vaginally applicable medicinal dosage form; vaginal films and membranes are also options. Pre-exposure prophylaxis against HIV infection has recently been researched, with an emphasis on the creation of pH-responsive systems.

For HIV prevention, scientists created a biphasic device based on TFV that combines organogels and hydrogels (bigels) that improved the way the polymer strands interacted with the vaginal mucosa as well as the enhancement of mucoadhesion properties and the duration of drug release. Due to increased hydrogen bonding between the carboxylic groups of pectin and the mucin glycoproteins of the vaginal mucosa, the high degree of esterification of pectin has added to the superior mucoadhesive properties of bigels. Because pectin is more soluble at greater pH, the bigels lost their structure more quickly in an SVF/SSF mixture than in SVF alone due to the acidic nature of pectin which led the substance to release rapidly [231].

Researchers developed a layered chitosan citrate and methyl polymethacrylate (Eudragit^®^ S100) to create a vaginal film based on tenofovir (TFV). Using polymer synergy, the polyelectrolyte complex’s stability and pH-dependent behavior were improved. Since ES100 and citric acid have crosslinked chitosan derivatives and allow polymer chains to migrate, the vaginal film was resistant and flexible. The film’s limited swelling capacity can be attributed in part to the crosslinkers’ creation of a 3D structure. Furthermore, the chitosan–mucosa interaction was enhanced by low water absorption and a high fraction of ES100. Moreover, SVF chitosan and ES100 crosslinking restricted water access in SVF and SVF/SSF combinations, which resulted in a longer half-life and slower drug absorption. Moreover, only chitosan in SVF/SSF released TFV, as ES100 solubilized at a higher pH in SVF/SSF. This also aided in a more rapid drug release. Further, no toxicity but only little damage was found in the mucosa after the addition of citric acid or ES100 in the preparations [232]. Formulation of two pH-responsive switchable formulations—the supramolecular polyurethane (PU) polymer and PU membrane—to improve siRNA delivery has been developed. The first study enclosed siRNA-loaded nanoparticles in PU hydrogel and physically crosslinked them within a segmented reservoir-intravaginal ring (IVR). In vitro release profiles showed close-to-zero release at pH 4.2 and steady release at pH 7.0. Copolymer chain physicochemical contact was pH-responsive. The use of 2,2-Dimethylolpropionic acid deprotonation at pH 7.0 produced a mild intermolecular hydrophobic interaction. Nanoparticles released quickly due to particle spacing and penetrated the mucus better with a close-to-zero zeta potential. The cell types on cytotoxic evaluation were unaffected by nanoparticles and hydrogel, respectively [233].

##### Enzyme-Responsive Delivery System

The antiretroviral drug maraviroc (MRV) was encapsulated in a HA/palm oil-based organogel to be used in HIV pre-exposure prophylaxis by using temperature and enzyme as stimuli [234]. Artificial neural networks modeled and optimized palm oil/hyaluronic acid-based organogel mucin absorption and flow for vaginal MRV delivery. The adjusted HAP organogel was viscous at pH 4.5, 399.1 mOsm/kg, and pH 5.9. The organogels had an average globule size of 581.8 ± 3.9 nm, and the in vitro release assay showed a release rate of 3.894 μg/cm^2^/min^1/2^ for the HAP organogel and 4.49 μg/cm^2^/min^1/2^ for the optimized formulation. The organogel’s thermosensitivity and HA sensitivity were achieved by incorporating hyaluronic acid and dicaprylate esters, and thermogelation occurred at 34.1 °C. The organogel released 2.5-fold more maraviroc when hyaluronidase was present than when it was absent. This is due to the fact that hyaluronidase is the first enzyme involved in the hydrolysis of the glucuronic acid groups of unbound HA [235,236]. Optimized organogel showed mucin adsorption and flow of 70.84% and 4.962 μg/cm^2^/min^1/2^, releasing MRV via temperature and HA. These findings suggest palm oil/hyaluronic acid organogel could give anti-HIV microbicides vaginally. This can guide HIV microbicide formulation trials. MRV organogel was non-cytotoxic for 14 days in vitro compared to untreated HeLa cells.

##### Temperature-Responsive Delivery System

There has been a rise in the number of suggested thermogelling systems in the literature to address the difficulties of VDDS. Thermogelling devices operate on the principle that a polymeric solution can transform into a gel when the temperature is increased. It is a common practice to make thermo-responsive hydrogels using the temperature-sensitive polymers chitosan and poloxamer. For this reason, many different drug-loaded thermo-responsive hydrogels have been developed for use in the therapy and prevention of vaginal diseases. The following parts detail research into thermo-responsive formulations for a variety of vaginal disorders.

**Figure 5:**
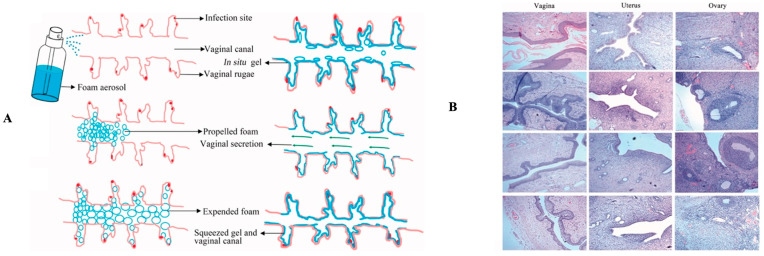
(**A**) A schematic diagram of the expanding thermal gelling foam aerosol as it expands, gels, and retains its shape. The foam aerosol form of ETGFA was used to deliver it to the vaginal canal; propellant was used to push the foam aerosol into the vaginal canal; the foam expanded and penetrated deep into the vaginal rugae; the foam thermally transformed into gel to cover the infectious sites on the vaginal mucosa; and the gel resisted self-cleaning during vaginal secretion and the contraction motion of the vaginal canal. (**B**) Rat vaginal, uterine, and ovarian tissue pathological sections (HE staining × 400). Either saline, blank ETGFA, or ETGFA were enriched with silver nanoparticles. Reprinted, free access from [237]. Copyright 2017. Taylor and Francis Journals.

To treat vaginitis and cervical erosion, Mei et al. devised an expansible thermal gelling foam aerosol (ETGFA) using P188, P407, and carbopol. Notably, the propeller-generated ETGFA foam with improved expansion height and duration time had improved formulation penetration efficiency and prevented foam liquefaction. Carbopol also improved the gel’s ability to adhere to the mucous membranes. Notably, the first hour was marked by a burst of drug release, followed by a more gradual release, and the loading dose was delivered via instant release (Figure 5A).

The MICs of the vaginal flora were comparable to the local drug concentration, and the Sprague Dawley rats had not been irritated by the addition of AgNPS to the ETGFA. The comprehensive irritation index of AgNP-loaded ETGFA was 2.66, while that of AgNPs was 3.25 (Figure 5B). Notably, AgNPs’ therapeutic effects were improved when they were administered in a foam and gel formulation [237]. A similar formulation was made with P407 and highly viscous chitosan (chitosan H), which had the highest viscosity and mucoadhesion values. As a result, BNZ-poloxamer hydrogel required less-frequent administration due to its high viscosity. Poloxamer hydrogel also improved mucoadhesive performance by adding high molecular weight chitosan to the formulation, as the chitosan maximized adhesion through entanglements and Van der Waal forces [238,239]. The formulation also had excellent hardness, compressibility, adhesiveness, cohesiveness, and elasticity. Specifically, a vaginal gel’s adhesiveness, cohesiveness, and elasticity are ideal for locating antimicrobial agents and allowing for full structural recovery after administration. Appropriate compressibility and hardness are also required [238,240]. Gupta et al. optimized a chitosan and gellan gum bio-adhesive in situ vaginal gel. As an ion-activated polymer, gellan gum acts as a bio-adhesive polymer and permeation enhancer. Clindamycin was used as a model drug because it is successful in treating bacterial vaginitis (BV). The formulation of 1% *w*/*v* chitosan and 1% *w*/*v* gellan gum had, thus, been chosen for further testing because it formed a firm, transparent gel upon administration [241]. The HET-CAM test results showed no irritation for 2 h (mean score 0). After 6 h, the mean score was 0.33, and after 24 h, it was 0.66 [241]. This study proved that combining chitosan and gellan gum could form an in situ vaginal gel with good bio-adhesion and retention properties while being non-irritating to the vaginal mucosa [241]. In addition, using gellan gum as the ion-activated gelling polymer and HPMC as the bio-adhesive polymer a different research investigation has been conducted to treat BV with an in situ activated gel and mucoadhesive strength combining both HPMC and gellan gum. However, in situ gelling could not be achieved with HPMC or gellan gum alone since they are not mucoadhesive agents. HPMC and gellan gum had, therefore, been significant mucoadhesion polymers and ion-activated gelling agents in the formulation [242]; the HET-CAM test results matched reference [241]. The in situ gel based on chitosan/gellan gum was non-irritating to mildly irritating, but it had been predicted that the vaginal mucosa would tolerate it well [241,242].

The current study examined the effect of bioresponsive polymers on drug release from a clindamycin gel formulation system for vaginal use. To prolong the effect of the bioresponsive system, alternative mucoadhesive polymers were added to the gel formulation. The formulation was tested in vitro for pH, viscosity, drug release profile, bio-adhesive force, spreadability, and stability. Simulated vaginal fluid was used as a diffusion medium to observe release. Carbopol, poloxamer, carrageenan, and other polymers can be used to successfully formulate clindamycin gel, but the choice of polymer and content determines viscosity and gel release. Therefore, the polymer combination effect was evaluated to prolong drug release from formulation, and the new formulation could replace traditional vaginal dosage forms [243]. The use of vaginal microbicides as a pre-exposure prophylactic for HIV/Aids also appears promising. Prior to the creation of a microbicide combining tenofovir disoproxil fumarate (TDF) and emtricitabine (FTC), experiments were conducted in silico and in vitro to evaluate their physicochemical qualities and biophysical effects in lipid model systems. Hydrogels were shown to be suitable carriers for TDF-loaded liposomes and FTC in these pre-formulation investigations. Therefore, it was decided to use zwitterionic liposomes and mucin at 41 °C and a mean diameter of 134 ± 13 nm to investigate the interaction. For HEC-1-A and CaSki cells, the chosen liposomal formulation might increase TDF penetration through polysulfone membranes (Jss = 9.9 µg·cm^−2^·h^−1^ per mL). Additionally, both liposomes containing TDF and hydrogels containing FTC best fit the Weibull model. These hydrogels also exhibited pseudoplastic profiles, making them acceptable for topical application. Therefore, TDF/FT vaginal co-delivery liposomal hydrogels may be a promising formulation in the long term [244]. To cure BV, research conducted on a thermosensitive in situ tinidazole gel. The use of P407 and E100 polymer followed by application uniformity were ensured due to a combination of spreadability and adherence. Notably, the formula’s mechanical qualities for vaginal gel was ideal. Similar to the ETGFA, the in situ tinidazole gel exhibited an initial burst, followed by a gradual release, of the drug. The anti-microscopic efficacy of the gel was superior to that of commercial products. However, in an HET-CAM test, the gel was shown to be somewhat irritating [245].

#### 4.2.2. Ocular/Eye Infection

Topical ocular administration was widely used to treat eye diseases including eye infections. However, the limited region of absorption, the lipophilic nature of the corneal epithelium, and a series of elimination processes such as nasolacrimal drainage, tear turnover, and tear evaporation reduce the contact time of medication with the corneal surface. Therefore, various innovative drug delivery techniques were tested for ocular delivery in recent years. Among these systems, in situ gels are notable as a lucrative technique for ocular drug delivery that is comparable to both conventional and new methods [246].

##### pH-Responsive Delivery System

The development of carbopol based-gel has been explored. Due to carbopol’s carboxyl groups which form an aqueous solution in an acidic condition, it was formulated in various carbopol combinations. In simulated physiological settings, carbopol (0.3%) combined with methylcellulose (1.5%) resulted in a formulation with low viscosity and compact gel [247]. The same group used carbopol and HPMC to construct a similar delivery mechanism. The results showed that a low-concentration carbopol in situ gel system can be used with cellulose derivatives without impairing in situ gelling behavior or viscosity [248]. Later, Srividya et al. attempted the same combination for the sustained delivery of ofloxacin. The system was constructed utilizing carbopol 940 (0.5%) and HPMC (Methocel E50LV) (1.5%), and it was evaluated for rheology, in vitro release, antibacterial activity, ocular irritation, and accelerated stability. The final formulation was reported to be therapeutically effective, stable, and non-irritating for up to 8 h [249]. Then, a once-daily ocular administration system for ciprofloxacin hydrochloride based on pH-stimuli in situ gelation was studied using a phase transition polymer (Carbopol^®^ 980NF) with a release retardant (Methocel^®^ K100LV) and a complexing agent (ion exchange resin). To avoid incompatibility with polyacrylic acid, ciprofloxacin was complexed with ion exchange resin. The created formulation was stable, non-irritating to rabbit eyes, and had a 98% drug release in vitro over 24 h [250].

The exploration of chitosan–poloxamer combinations (poloxamer F127 and poloxamer 188) still continues to use ciprofloxacin hydrochloride as a drug model. The rheological behavior and release kinetics of a mixture of pluronic (15%) and chitosan (0.1%) showed a *Newtonian* flow in non-physiological settings (pH 4 at 25 °C) but transformed to a pseudoplastic under physiological conditions (pH 7.4 at 37 °C). In physiologic and non-physiologic settings, larger concentrations of pluronic (25%) also displayed a pseudoplastic flow, indicating unsuitability for the manufacture of these systems. However, increasing the chitosan concentration or molecular weight slowed the release rate by reducing gel penetration. Moreover, the Fickian or diffusion mechanism was reported to be independent of the components in all gel combinations [251].

##### Enzyme-Responsive Delivery System

Polypeptide organizations may benefit from the use of cage-shaped nucleic acid nanocarriers. However, the demand for simple loading mechanisms that faithfully mimic nature’s host–guest systems to induce encapsulation of antimicrobial peptides (AMPs) without compromising their biological function remains unfulfilled. DNA nanogels that can rapidly load L12 peptide in situ prior to or during thermal annealing was formulated. The research is able to keep the AMPs within the DNA nanogel by taking advantage of their binding affinity to L12 in the polyanionic core. The result showed that the pre-loaded L12 nanogels had excellent encapsulation effectiveness, minimal toxicity, and sustained drug release translated to potent antibacterial action, even at high temperatures. Using *Staphylococcus aureus* as a model of infectious bacterial keratitis, it was found that both clinical symptoms and bacterial bioburden resolve rapidly. In addition, endogenous enzymes (such as DNases) in the ocular fluid and infection environment (such as *S. aureus*) can aid in the slow and steady release of encapsulated antimicrobials. This research has broad implications for combating the alarming increase in resistance since it paves the way for the creation of DNA nanocarriers for caging AMPs [252]. Due to its low drug toxicity and great ocular tolerability, the nanogel-loaded antimicrobial peptide approach is a promising approach for the clinical treatment of bacterial keratitis [253].

##### Temperature-Responsive Delivery System

The development of dual pH/temperature-responsive sodium alginate/pNIPAAm hydrogel loaded with antibiotic oxytetracycline (OTC) for controlled drug release showed a good properties [254]. Notably, the swelling ratio reached its maximum at pH 10.9 when the carboxyl groups in SA and acrylic acid (AA) moieties ionized, causing an increase in electrostatic repulsion [255]. Cell cytotoxicity and antibacterial properties of oxytetracycline (OTC)-enclosed, dual-responsive sodium alginate and N-isopropylacrylamide hydrogels (SA/pNIPAAm) were examined. Different acid-base circumstances and temperature conditions were investigated for their effects on the molecular OTC release. The hydrogels’ IC50 values for three consecutive days were 50.11 μg mL^−1^, 34.27 μg mL^−1^, and 22.39 μg mL^−1^, demonstrating outstanding time-dependent antimicrobial efficacy. Since the polymer is so sensitive to temperature, the SA/pNIPAAm hydrogel has a temperature sensitivity of its own. Therefore, as long as OTC levels remain within the therapeutic range for the first 48 h, a slow but consistent release is to be expected. Furthermore, when raising the OTC-loaded SA/pNIPAAm hydrogel concentration from 1 µg·m^−1^ to 100 µg·m^−1^, the Gram-positive bacteria *E. coli* viability was reduced. A test using SA/pNIPAAm hydrogel loaded with OTC revealed good cell survival, confirming the hydrogel’s ability to act as a biocompatible vehicle for the regulated delivery of OTC. In addition, SA/pNIPAAm which is sensitive to both pH and temperature may improve the selectivity of drug release at the best possible spot [254].

A dual-stimulated, thermo/pH-responsive in situ nanogel for the delivery of ciprofloxacin was developed by combining the thermosensitive polymer PNIPAAM with the pH-sensitive polymer methacrylic acid (CIP). In an initially transparent solution, the in situ gel changes into a gel at pH 7 and at 36 °C to extend the topical delivery of CIP, as demonstrated by this in situ nanogel. Additionally, the effect of this new gelling mechanism on bacterial proliferation and the evolution of drug resistance was studied. The antibacterial activity of nanogels that release CIP was tested at the minimum inhibitory concentration, and the results showed promise for topical use in the treatment of bacterial keratitis [256].

Known as a lipophilic drug, itraconazole absorption for ocular administration was limited. The purpose of this study was to incorporate itraconazole into a nanocrystalline carrier system with the stabilizer Pluronic^®^ F127, which was then formulated into a thermosensitive in situ ocular gel. When compared to a free itraconazole suspension in water, the nanocrystals demonstrated superior in vitro activity against *Candida albicans* (CA). Ex vivo ocular-kinetic studies on infected porcine eye models revealed that the optimized formula of thermosensitive in situ ocular gel had a better profile than the standard gel base and revealed a 93% reduction in the CA population after 48 h. Overall, this research has demonstrated a novel approach to developing more advanced treatments for fungal keratitis [257].

### 4.3. Respiratory Tract Infections

Enhancing the delivery of antibiotics straight to the lung appears to be plausible in the treatment of respiratory infections. However, direct administration of the antibiotic to the infection site was required to achieve better, more effective drug concentrations in certain organs, such as the lung. This approach’s goals have been to use lower doses of medication, avoid or reduce systemic exposure, and reduce systemic adverse effects. Today, more research has been done on inhaled nanopharmaceuticals to provide targeted distribution and reduce antimicrobial resistance [258,259].

#### 4.3.1. Tuberculosis

##### pH-Responsive Delivery System

The effectiveness of treating *M. tuberculosis* could be considerably improved by using a nanoparticle drug delivery technology that targets and delivers high quantities of medication directly into *M. tuberculosis*-infected cells. For example, the design of 100 nm and 50 nm stimuli-responsive mesoporous silica nanoparticles as prodrug carriers for the key anti-tuberculosis drug isoniazid. The drug is bound to the aldehyde-functionalized, PEI-PEG-coated nanoparticle through hydrazone bond formation. When the pH of the endolysosomal fluid drops below a certain threshold, the drug is released from the nanoparticles. It showed that human macrophages infected with *Mycobacterium tuberculosis* readily consume isoniazid-loaded PEI-PEG-coated nanoparticles, which then kill the intracellular bacteria in a dose-dependent form. The nanoparticles were also safe and far more effective than the free drug in a rat model of pulmonary tuberculosis [260].

Using the clay mineral “montmorillonite” as a nanocarrier, a new formation of a hybrid and pH-responsive system for rifampicin, using different factorial designs to determine how operational variables affected the incorporation of the drug. The experimental conditions were such that a drug dose incorporation of 98.60 ± 1.21 mg/g was possible. About 70% of the drug was released after 16 h in simulated intestinal fluid, proving the efficiency of the pH-dependent method developed through in vitro release trials. Rifampicin is slowly released from montmorillonite, as shown by fitting the experimental release data to the theoretical model developed by Higuchi and Korsmeyer-Peppas. Understanding how this raw clay interacts with the medicine makes it more promising as a potential carrier in the creation of a physically and chemically stable anti-TB/clay hybrid system [261].

##### Enzyme-Responsive Delivery System

Pulmonary drug distribution was achieved by using poly(ethylene glycol) hydrogel microparticles. The size of hydrogels can be tailored for administration into the bronchi and swell to evade uptake and clearance by alveolar macrophages, making them ideal for pulmonary delivery. A solution polymerization approach was devised to make enzyme-responsive hydrogel microparticles for pulmonary administration. This approach makes spherical poly(ethylene glycol) (PEG) microparticles from high-molecular-weight poly(ethylene glycol) diacrylate (PEGDA) precursors with peptides. The synthesized hydrogel microparticles disintegrate when matrix metalloproteinases are overexpressed in pulmonary disease. PEGDA dissolved in water at high concentrations produced small hydrogel microparticles suitable for inhalation from solid precursors. Depending on the precursor polymer’s molecular weight and water content, the particles’ average diameter was found at 2.8–4 μm. It was also observed that the mesh size of the particles had a direct correlation with the rate of their enzymatic breakdown [262].

**Figure 6:**
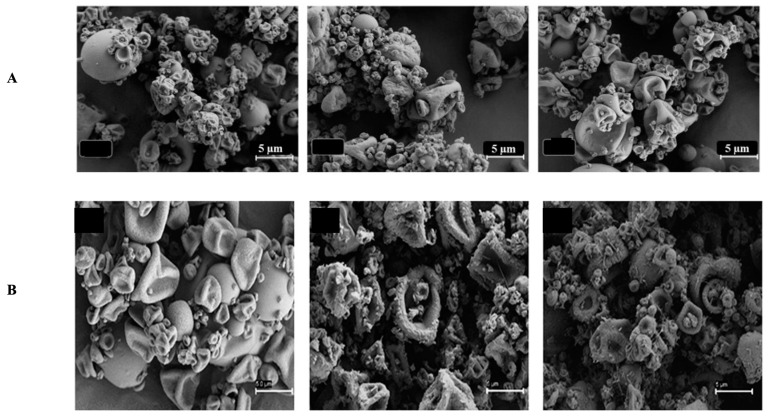
(**A**) Scanning electron microscopy images of locust bean gum-based microparticles. The locust bean gum microparticles were unloaded, locust bean gum:isoniazid = 10:1 (*w*/*w*) microparticles, locust bean gum:rifabutin = 10:0.5 (*w*/*w*) microparticles, and locust bean gum:rifabutin = 10:0.5 (*w*/*w*) microparticles, which were indicative of formulations with varied levels of rifabutin. Reprinted with permission from [263]. Published by MDPI 2016. (**B**) SEM microphotographs of konjac glucommanan-based MP; konjac glucomannan/isoniazid/rifabutin (10/1/0.5, *w*/*w*); konjac glucomannan-Man/isoniazid/rifabutin (10/1/0.5, *w*/*w*); and konjac glucomannan-Leu/isoniazid/rifabutin (10/1/0.5, *w*/*w*). Scale bar = 5 μm. Reprinted with permission from [265]. Copyright 2019 Elsevier Inc.

In the alveolar zone of the lung, these bacteria grow in alveolar macrophages [263]. There are galactose and mannose residues in locust bean gum (LBG), a polysaccharide that can be used to enhance the phagocytosis of macrophages. In this study, for the first time, spray-dried LBG microparticles comprising either isoniazid (INH) or rifabutin (RFB), first-line antitubercular medicines, have been described. This is because the theoretical properties of microparticles (aerodynamic diameters between 1.15 and 1.67 m) are suitable for deep lung administration. It has been found that RFB-loaded microparticles are toxic to lung epithelial cells (A549 and THP-1 cells) and macrophages (THP-1 cells), but, notably, these concentrations were quite high compared to in vivo settings. Macrophages also phagocytosed a high percentage of LBG microparticles (>94%) in this study. Additionally, LBG microparticles, either in the presence or absence of a drug, had presented a convoluted surface (Figure 6A). Furthermore, after MTT evaluation, it should be noted that the released enzyme amount was similar to or even lower than that seen in the control group (cells incubated with culture medium). Therefore, the evidence gathered so far had proven that the tuberculosis therapeutic system shows great promise [264].

Many studies had used nanocarriers for the treatment of tuberculosis, some of which focused on lung delivery applications. Various actions were taken, including spray-drying, which was proposed as a technique for producing konjac glucomannan microparticles (KGM) to be used in lung tuberculosis therapy. The inhalation of microparticles containing an association of INH and RFB, which are considered two first-line therapies for tuberculosis, was developed. The effects of mannitol and leucine as spray-drying excipients on drug-loaded KGMs have been tested. Notably, the MP yields ranged from 69 to 79 percent, which is considered satisfactory. An example of KGM-based MP is shown in Figure 6B. Notably, when excipients are used in the manufacture of inhalable microparticles to treat tuberculosis, their irregular shape may help minimize the forces of contact and cohesiveness between the particles, which could lead to better dispersion and lung deposition [265].

**Figure 7:**
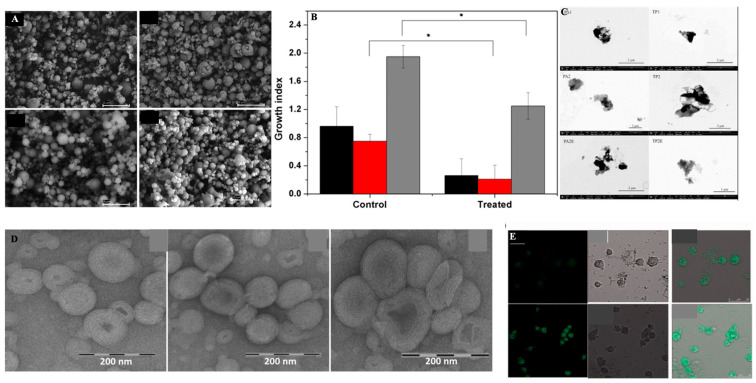
(**A**) SEM images of mannitol microspheres with the inclusion of glyceryl dibehenate solid lipid nanoparticles and glyceryl tristearate solid lipid nanoparticles, as well as trehalose microspheres with the inclusion of glyceryl dibehenate solid lipid nanoparticles and glyceryl tristearate solid lipid nanoparticles (scale bar: 20 μm). (**B**) The effect of the pulmonary administration of rifabutin-glyceryl dibehenate solid lipid nanoparticles microencapsulated in mannitol on the growth index of *Mycobacterium tuberculosis* in BALB/c mice’s liver, spleen, and lung (in black, red, and gray, respectively). Statistical analysis between the control (infected and nontreated) and treated groups, for each organ was performed using one-way ANOVA with Dunnet’s post hoc test (*, *p* < 0.05). Permission/license was granted by ACS Publication 2017 [267]. (**C**) TEM images of rifampicin lipid nanoparticle assemblies. Reprinted with permission from [267]. Copyright 2017 Elsevier Inc. (**D**) Negative electron transmission micrographs of rifampicin-loaded glycerosomes, HY-glycerosomes, and TMC-glycerosomes. Reprinted with permission from [268]. Copyright 2016 Elsevier Inc. (**E**) Confocal laser scanning microscopic images showing the endocytosis of the nano-emulsion in macrophages (fluorescent micrographs, differential interference contrast images). Copyright 2017. Taylor and Francis Journals [269].

Rifampicin (RIF)-loaded lipid NP assemblies (SLNas) with new mannose derivatives for inhaled anti-tuberculosis therapy via a dry powder inhaler (DPI) are being developed on a regular basis. Since MRs are overexpressed on the membranes of infected alveolar macrophages (AM), which are *Mycobacterium tuberculosis*-favored sites of infection, mannose is regarded as a relevant ligand for active drug targeting. All of the SLNas samples contained micro-aggregates of well-formed NPs with a round flaky shape and a corrugated surface, as evidenced by circularity values ranging from 0.56 to 0.63 *(p* > 0.05), with a value of 1 indicating a spherical shape. Since the macrophages, the bacteria’s host cells, have numerous therapeutically relevant surface receptors, this strategy is applicable [266]. However, any strategy for treating tuberculosis via lung delivery must take into account the fact that macrophages inside the alveoli house all bacteria. To achieve this, SLNs were proposed as a carrier. Encapsulated in microparticles of mannitol, the RFB-loaded microparticles (100 nm glyceryl dibehenate) were able to achieve the zone inside the alveolar, with 44% of particles with a size under 6.4 nm. The mannitol and trehalose microspheres loaded with SLN are shown in Figure 7A. However, some of the mannitol microspheres had slightly convoluted morphologies in the general microsphere shape. Nevertheless, using the *Mycobacterium tuberculosis* strain H37Rv as a murine model of infection showed an effective antibiotic delivery to the lung as well as liver and spleen distribution. Antibacterial activity was also found to be improved in the treated animals compared to the control group (Figure 7B) [267].

RIF was included in this SLN formulation, which had comprised palmitic acid and cholesteryl myristate. However, no significant differences in PDI values were found among the samples *(p >* 0.05) that contained NPs in a water suspension before freeze-drying (sizes ranged from 0.39 to 0.41 μm). Figure 7C shows that freeze-dried SLNs derived from nanoparticle aggregates had an irregular shape, as confirmed by the circularity values that ranged between 0.45 and 0.69. The size range of SLN’s main population was ranged from 0.72 ± 0.02 µm to 1.38 ± 0.19 µm and PDI values were from 0.54 ± 0.09 to 0.81 ± 0.21. The SLNs had also been slightly smaller (0.72 0.02 m and 0.80 0.15 m, respectively) when ethanol was used as a co-solvent (PA2E and TP2E samples) but the PDI values had not been significantly different. Notably, minorities (less than 15%) had a diameter of 0.30 to 0.42 nm (Figure 7C). Afterward, it was freeze-dried to produce an inhaled powder. FPFs between 30% and 50% were found to be within the mass median aerodynamic diameter (MMAD) range of 5–7 nm. Improvement in alveolar macrophage passive targeting, sufficient payloads (10–15%), and possible drug maintenance in the respiratory system before macrophage internalization had, therefore, been achieved through the mannosylation of the SLN. Furthermore, despite respirability being hampered by powder cohesiveness, surface mannosylation had caused quicker macrophage phagocytosis, demonstrating indications of an active targeting promotion in comparison to non-functionalized SLNs [263,266].

Antitubercular drug rifampicin was chosen to encapsulate with polymer-glycerosomes, to achieve a more stable particle rather than the use of conventional liposomes. TMC-glycerosomes were prepared by combining phospholipid/glycerol with trimethyl chitosan (TMC) or sodium hyaluronate (HY) to form TMC-glycerosomes. After nebulization, the MMAD was found at 4.0 ± 1.2 while the FPF value was up to 77% [270]. Based on TEM analysis, all dispersions had closed lamellar structures. Glycerosomes were spherical multilamellar vesicles. HY and TMC glycerosomes were also multilamellar and spherical, but slightly aggregated (Figure 7D). In any case, carriers outperformed free drugs in terms of aerodynamics, and drug incorporation in vesicles increased efficacy against *Staphylococcus aureus*. In rats, glycerosomes increased rifampicin accumulation in the lungs, but not in other organs. It was concluded that the hyaluronic acid formulation performed better [268].

The units of hyaluronic acid’s (N-acteylglucosamine) were well known to interact and be well attached with the CD44 and the mannose receptors [271,272,273]. Nebulized oleic acid-based nanoemulsions loaded with rifampicin were tested for satisfactory respirability with FPF in the range of 62–73%. Chitosan and folate may have boosted cell internalization by interacting well with macrophages, which may have led to an increase in cell internalization. While the drug concentration in the lungs was higher than the concentration in the plasma, all nanoemulsions demonstrated over 95% aerosol emission and over 75% inhalation efficiency in an in vivo test. The fluorescence intensity of macrophages incubated with nanoemulsions was greater. This was proof of nanoemulsion absorption by cells. Second-generation nanoemulsion coated with chitosan showed considerably higher internalization than that of the third-generation nanoemulsion decorated with chitosan-folate conjugate after 2 h (Figure 7E) [269]. Chitosan nanoparticles produced by ionic gelation using tripolyphosphate have also been proposed for antitubercular drug delivery. Leucine and lactose were employed in the initial trial to attain an FPF of 45% [274]. Similar nanoparticles were recently linked to bedaquiline (size found to be varied within 70 and 700 nm according to the preparation conditions). In comparison to the control DPI formulation, the nanoparticles freeze-dried to a powder and registered a 28% FPF and a 3.38 nm MMAD (15 percent FPF and 4 nm MMAD). Inhalation of the microencapsulated nanocarriers increased medication concentrations in the lungs of rats, according to the research, which revealed no damage to the nanoparticles [275]. The use of chitosan with positive charges (from amino groups) interacts with the negatively charged surface of macrophages, resulting in beneficial consequences, according to the authors of the second study. Even while macrophages have a great affinity for chitosan, most studies omit to indicate that this may be owed to macrophage surface receptors being able to identify the polymer’s N-acetylglucosamine units [273,276]. They were freeze-dried to form powdered carboxymetylchitosan nanoparticles loaded with isoniazid and rifampicin. Following inhalation by rats, carriers accumulated more drugs in the lungs than free drugs. In addition, drugs stayed longer in the lungs, while the other organs (liver and kidney) had lower drug composite levels [277].

##### Temperature-Responsive Delivery System

After long-term use, isoniazid (INH), a first-line bone tuberculosis (TB) treatment, has serious adverse effects. Nano-drug delivery technologies for INH administration are promising; however, inefficient drug accumulation in infected areas limits therapeutic efficacy. A novel liposome-in-hydrogel system for localized bone TB treatment using local administration to obtain high drug concentration at focal locations with minimum systemic exposure has been developed. D-INH, a hydrophobic derivative of INH with improved activity and biosafety, was loaded to sustain drug release. Phase transition test and rheological investigations showed that the hybrid system was thermo-responsive and self-healing, making it appropriate for intra-articular injection. In vivo microdialysis showed that the method can rapidly release drugs into the synovial fluid to reach inhibitory quantities following localized injection, followed by steady-state drug release. In vivo optical imaging studies showed a prolonged drug release profile. This bone TB drug delivery method is a promising approach

##### Light-Responsive Delivery System

The development of a dual therapeutic strategy involving photothermal (PT) and antibiotic treatment for the eradication of bacteria are both delivered by a nano-assembly that is triggered by a near-infrared (NIR) laser, the combination is then called PTT. When used together, the treatments have a multiplicative effect on the patient’s recovery. Safe antibiotic delivery and localized release near the bacterial surface lower non-specific side effects and maximize the therapeutic benefit. The nanoassembly’s bedaquiline (BDQ)-carrying mesoporous silica nanoparticles (MSNPs) are encapsulated within NIR active gold nanorods (GNRs). The mycobacteria-targeting peptide was conjugated to a thermo-sensitive liposome (TSL) and then coiled around the assembly. The ultimate nanoassembly’s adhesion to the mycobacterial surface wass mediated by NZX (GNR@MSNP@BDQ@TSL@NZX). NIR laser irradiation caused GNRs to transform the laser’s photo energy into localized heat, which in turn caused TSL to melt and release BDQ. The finished nano-assembly was 20 times more effective than the free drug equivalent in inhibiting the growth of *Mycobacterium smegmatis* (Msmeg). Lung cells harboring mycobacteria could also have their development stunted by the completed nano-assembly [26].

#### 4.3.2. Cystic Fibrosis

Cystic fibrosis (CF) allows opportunistic infections to enter the lungs. Mutations in the CFTR gene are thought to be the cause of this hereditary condition. The protein encoded by this gene can be found in the membranes of epithelial cells. Mucus secretion and buildup in the airways can be caused by genetic malfunction, which can lead to protein damage and bronchial blockage. The most frequent bacteria detected in mucus are *Pseudomonas aeruginosa* and *Staphylococcus aureus* [252,253,278,279,280]. This justifies the use of antibiotics alongside bronchodilators and mucolytics in cystic fibrosis treatment.

##### pH-Responsive Delivery System

*Pseudomonas aeruginosa* biofilms in the airways are the leading cause of recurrent lung infections in CF patients due to their increased resistance towards medicines, and it has been found that PEGylating tobramycin improves the drug’s mucus penetration and its efficacy against these biofilms. When compared to free tobramycin, PEGylated tobramycin significantly slows the growth of bacterial biofilm after 24 h of treatment. PEGylated antibiotics have a greater antibiofilm impact than unmodified antibiotics after only 5 h of therapy in a mucus barrier model of cystic fibrosis. The increase of thickness, increase of solid content, acidic pH, and heavy dehydration all work against medication transport in cystic fibrosis mucus [281]. The PEGylated contains a pH-responsive imine bond that accelerates the drug release in acidic mucus environment [282]. Cystic fibrosis mucus, in a similar fashion, traps aminoglycoside drugs such as tobramycin. PEGylated tobramycin’s increased biofilm suppression indicates indirect evidence of the drug delivery system’s mucus penetrating characteristics in cystic fibrosis mucus [283].

Dry powdered Tobramycin was the first licensed aerosolized antibacterial agent for *Pseudomonas aeruginosa*. However, due to this drug’s weak mucus penetration, fast clearance, and low concentrations at the infection site, it is inefficient in avoiding bacterial infection-related consequences [284]. Cystic fibrosis researchers are working to develop more effective treatments. This could be aided by nanotechnology. A severe problem is the penetration of mucus. Infections can be eradicated more effectively with better medicine delivery if this barrier is removed. Nanoparticles coated with PEG (polystyrene coated with polyethylene glycol—PEG) of diameters up to 300 nm exhibit higher retention and more uniform dispersion in the lung than similar-sized nanoparticles without PEG and so are mucoadhesive [285]. The encapsulation of Colistin into PLGA nanoparticles was done by a spray-drying process, and the mixture had sufficient aerodynamic qualities. Lactose yielded an MMAD of less than 5 nm, while mannitol yielded an MMAD of 8 nm. Colistin’s anti-biofilm action was found to be enhanced in experiments using chitosan-modified particles, possibly as a result of the particles’ capacity to penetrate biofilms and maintain the drug release [286]. Nanoparticles modified with PVA had previously been shown in vivo to reach the alveoli, while those changed with chitosan were found in the upper airways, probably as a result of their unique aerodynamic features [287]. Concerning microencapsulating ciprofloxacin, we employed a combination of nanoparticles made of PEG-g-phthaloyl chitin and alginate microparticles that swelled when heated. After IT administration to rats, greater amounts of the encapsulated molecule were observed in lung tissue and lavage fluid than in a physical mixture of lactose and micronized medicine [288].

##### Enzyme-Responsive Delivery System

*Pseudomonas aeruginosa* (PA), an opportunistic pathogen, causes severe lung infections in immunocompromised people. Oral administration of high amounts of small-molecule antibiotics for bacterial infections causes off-target toxicity and drug resistance. Antibiotic delivery systems that target bacterial infections would lower antibiotic use. Colistin (Col), an antibacterial peptide, is the last option for multidrug-resistant MDR-PA. Drug-loaded mesoporous silica (MSN) core, liposomal layer, and PA-targeting LL-37 peptide make up the nanoassembly. The liposomal shell stops drug release before the nanoassembly reaches the bacteria. PA-excreted lipase breaks down the liposome bilayer, with Col release profile at 90% within 40 h compared to 75% release after 80 h (non-bacteria). The formulation had 6.7-fold greater antimicrobial activity than free Col. The nanoassembly targeted and blocked lung epithelial cell intracellular PA14 bacterial strain and showed only 7% lung epithelium cell viability. A non-cytotoxic effect was observed. Thus, this lipid-coated targeted nanoassembly can release antibiotics and enable a safe delivery of antimicrobials [289].

##### Temperature-Responsive Delivery System

**Figure 8:**
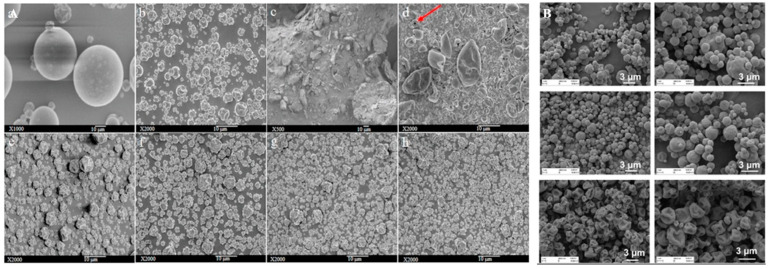
(**A**) This SEM images consist of (**a**) supplied kanamycin; (**b**) spray-dried kanamycin; (**c**) supplied l-leucine; (**d**) spray-dried l-leucine (red arrow indicates the pore); (**e**) spray-dried kanamycin with 5% *w*/*w* l-leucine; (**f**) spray-dried kanamycin with 10% *w*/*w* l-leucine; (**g**) spray-dried kanamycin with 15% *w*/*w* l-leucine; (**h**) spray-dried kanamycin with 20% *w*/*w* l-leucine. Reprinted with permission from [294] Copyright 2017 Elsevier Inc. (**B**) SEM micrographs of multifunctional DPI formulations. Azi/NAC, Cipro/NAC, and Tobra/NAC. Reprinted with permission from [293]. Copyright 2019 Elsevier Inc.

Co-amorphous and polymeric amorphous solid dispersions (CASDs and PASDs) are two examples of newer formulation techniques for solid molecular dispersions. Crystalline medicines can be amorphized and stabilized with a tiny molecular weight conformer using CASDs. In many cases, the high glass transition temperature and strong intermolecular hydrogen interactions make these systems remarkably stable [290]. CASDs are thought to improve drug kinetic bonding and apparent stability by increasing glass transition temperature solubility and decreasing amorphous state energy [291]. There are established conformers for forming co-amorphous solid dispersion. As a conformer with anti-hygroscopic properties and the ability to produce coarse particles, L-leucine is frequently used in studies aimed at improving the aerosolization properties of particles. There were inhalable (6.1 m) spray-dried powder particles, as shown in Figure 8A. It was found that the L-leucine powders were wrinkly, amorphous, and dry. Tolerance was shown for the powders in respiratory cell lines, which remained stable at 25 °C and 15% relative humidity. The best improvement in aerosolization properties was seen with the 5% L-leucine formulation [292].

Researchers used a mucolytic amino acid derivative known as L-leucine and n-acetyl cysteine (NAC) to spray-dry azithromycin (AZI), tobramycin (Tobra), and ciprofloxacin (Cip). These DPI formulations were found to be amorphous after spray-drying. Co-amorphous solid dispersions (CASDs) formed by Azi/NAC and Tobra/NAC can tolerate stress storage. Inhalation of the resulting mass median aerodynamic diameters was found to be safe (5.0 m). Azithromycin and n-acetyl cysteine alone reduced biofilm formation by 25%, while multifunctional antibiotic/NAC formulations maintained or increased antibiotic susceptibility. The efficacy of treatment can be improved while formulation properties are improved by including mucolytics such as NAC into CASD conformers. All three antibiotic/NAC combinations were gathered and stored in the same way. A low moisture content and a low aggregation tendency are inferred from this observation. All formulations comprised primarily spherical particles, as shown in Figure 8B. The surfaces of the Cipro/NAC and Azi/NAC formulations were slicker than those of the Tobra/NAC only. Particle cohesiveness is reduced and aerosolization is increased when L-leucine is added, as previously indicated [293].

Although other researcher had already formulated spray-dried azithromycin with varied amounts of L-leucine, the powder shape and FPF (*p* > 0.05) did not alter significantly. The in vitro disintegration rate increased, while the percentage of emitted dose remained the same (*p* > 0.05). Particle surface morphology and composition explain the lack of change in FPF and emitted dose. The more corrugated a particle surface is, the better it aerosolizes. This is due to L-leucine’s ability to improve aerosolization [295].

Dry powder microparticles of ciprofloxacin complexed with calcium or copper ions (Cip-Ca and Cip-Cu) was created using spray drying method [296,297]. Using typical spray-dried morphology and in vitro characteristics, the powders produced were amorphous with shell-like structures. Transcellular diffusion from lung fluid can actually be slowed by the creation of metal counterions. Epithelial fluid Cip-Cu had five times as much ciprofloxacin as plasma Cip-Ca. An increase in Cip-Cu stability, as well as slower plasma absorption and a longer elimination half-life, was connected to the stronger complex produced. In order to maximize antibiotic lung retention, it is necessary to choose the right metal ion [296].

In order to create mucus-penetrating nanoparticles, a synthesized of compritol-based solid lipid nanoparticles (SLNs) coated with a hydrophilic sheath consisting of poloxamer, Tween, and PVA has been evaluated. The resulting nanoparticles had mean sizes of 81, 155, and 215 nm, respectively, and an overall negative zeta potential. Based on the results of an in vitro mucus penetrating analysis, nanoparticles coated with poloxamer only needed to be incubated for 2 h to penetrate the mucus layer and reach the underlying epithelia, while nanoparticles coated with Tween and PVA needed 4 h. Longer incubation times, between 6 and 24 h, allow nanoparticles to get through the mucus layer and into the Transwell membrane underneath. Nanoparticles coated with poloxamer diffused the fastest via CF sputum, followed by those coated with Tween and PVA. Nanoparticles’ detectability close to the mucus network provides more proof of their mobility. Nanoparticles coated in Tween appeared to be rapidly absorbed by the cellular monolayer underlying the mucus network, as evidenced by their staining in the cytoplasm [298]. Researchers Alp et al. looked at the biophysical interactions that occur between particles made of lipids that can penetrate mucus and the mucus layer in the lungs. The stearic acid-based SLNs included the emulsifier Pluronic F127 and the co-emulsifier non-ionic surfactant Tween 20. In cystic fibrosis, the mucin network’s average pore size decreases from 400 nm in healthy persons to 140 nm, and its thickness also increases. When the mucus layer was 2.6 mm thick, only about 28% of the SLNs with sizes between 35 and 100 nm were able to penetrate it, but this increased to 36% when the thickness was lowered to 1.2 mm. Mucolytic agents, which chemically and physically disturb the mucus layer, were not used in the penetration of reported SLNs [299].

##### Redox-Responsive Delivery System

DPI formulations of polymeric cyclosporine drug nanoparticles (1–100 nm) are found to be strongly aerosolized [300]. The surface charge of modified polymer drug nanoparticles enhances drug delivery to diseased lungs [301]. Due to the small samples size, Salvati et al. was unable to assess the cytotoxic effects of 1,2-dipalmitoyl sn glycero-3 phosphocholine and 1,2-dipalmitoyl glycero-3 (phosphor-rac-1-glycerol) sodium salt. Researchers observed that polymeric drug nanoparticles (cationic charged/neutral) attach to the mucus layer of the lungs and have long-lasting drug release capabilities [294]. It was found that inhaled nanoparticles could change drug interactions with target cells [302].

Cystic fibrosis patients would benefit from taking ciprofloxacin-loaded lipid core nanocapsules (LNCs) because of their ability to penetrate mucus and kill bacteria living in secretions. The oleic acid-based lipid core increased ciprofloxacin solubility thanks to its lipophilic characteristics. In addition, ciprofloxacin’s -COOH and -NH2 functionalities showed ionic chemical interactions and hydrogen bonding with oleic acid, which guarantees a greater drug loading. The mucus penetration of LNCs was increased by 50% compared to the drug suspension, suggesting that their average size was below 200 nm. Initial drug release from LNCs was 66% after 4 h, primarily due to free diffusion, while subsequent drug release from LNCs was 30% after 20 h, demonstrating regulated drug release. In the simulated lung fluid, the LNCs released 96% of their drug load after 24 h, while the ciprofloxacin solution demonstrated a burst release of 97.9% after only 4 h. Intriguingly, half of the ciprofloxacin in the solution was released in less than 60 min, while it took the LNCs 150 min to do the same. Therefore, the LNCs’ ability to achieve prolonged release justifies significantly reduced dose frequency and enhanced patient compliance [303].

##### Light-Responsive Delivery System

Phospholipid-decorated gold nanorods (DSPE-AuNR) suspension was studied for its photothermal-induced bactericidal activity against *Pseudomonas aeruginosa* in both planktonic and biofilm cultures. Planktonic *Pseudomonas aeruginosa* cultures treated with a DSPE-AuNR suspension (0.25–0.03 nM) and then, when exposed to a continuous laser beam, experienced a reduction in viable bacteria of around ~6 log cycles compared to the control. After being exposed to laser excitation with varying doses of DSPE-AuNR, the viable count of *Pseudomonas aeruginosa* biofilms dropped by a factor of ~2.5 to 6.0 relative to the control. *Pseudomonas aeruginosa* biofilm viable count was reduced by ~4.5–5 log cycles due to the photothermal ablation activity of DSPE-AuNR (0.125 nM), which injected into poloxamer 407 hydrogel. The morphology of the bacterium was also significantly altered, and the cell membrane was lysed according to TEM images of the bacteria that had been subjected to photothermal treatment, as compared to untreated bacteria. The findings also showed that the photothermal-induced bactericidal activity was similar between the continuous and pulse laser beam types [304]. Nanoparticles having a range of physicochemical properties have also been found to interact with and penetrate into biofilms. Mucus penetration and interaction with a *Pseudomonas aeruginosa* cystic fibrosis bacterial biofilm model were shown to be enhanced by a lipid shell-enveloped polymeric nanoparticle with a high integrity of lipid shells [305]. In conclusion, *Pseudomonas aeruginosa* biofilm could also possibly be eradicated using the phospholipid-coated gold nanorod nanoplatform in cystic fibrosis patients.

### 4.4. Gastrointestinal Infection

#### 4.4.1. pH-Responsive Delivery System

**Figure 9:**
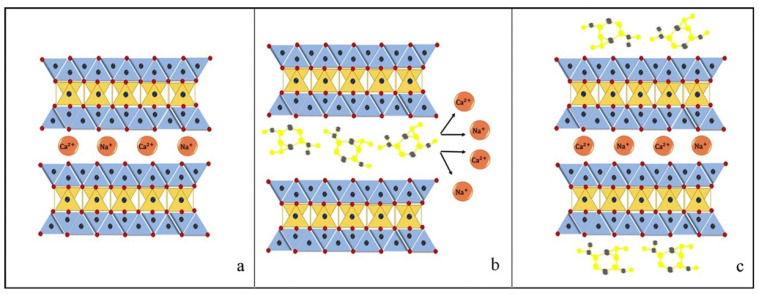
Adsorption mechanism of tetracycline on montmorillonite ((**a**) montmorillonite before adsorption of tetracycline, (**b**) cation exchange mechanism adsorption, and (**c**) adsorption of tetracycline onto external surface by complexation). Reprinted with permission from [307]. Copyright 2019 Elsevier Inc.

Antimicrobial drugs used to treat *Helicobacter pylori* (*H. pylori*) infections are rendered ineffective by the stomach’s acidic environment. *H. pylori* is poorly targeted by antibiotics, resulting in an imbalance in the gut flora. *H. pylori* may be eradicated more effectively and antibiotic side effects could be reduced if oral medicine delivery systems have improved stability at low pH and higher specificity to target *H. pylori*. The UCCs-2 and PLGA were used to synthesize pH-sensitive amoxicillin-loaded AMX-PLGA/UCCs-2 nanoparticles for the targeted treatment of *Helicobacter pylori* infection using UCCs-2 as a targeting moiety. The nanoparticles were made through the evaporation of two emulsions in the presence of a solvent. We employed an orthogonal design to create a pH-sensitive medication delivery device. The study was conducted both in vitro and in vivo. AMX-PLGA/UCCs-2 nanoparticles are an effective UreI-mediated targeted drug delivery device for anti-*H. pylori* treatment, according to the cell absorption process [306]. The development of pH-responsive montmorillonite nanosheets complexed with tetracycline showed isoelectric point around 5.5, which means that it is positively charged at a pH of 2.4, then the montmorillonite interlayer spaces are positively charged, and the tetracycline is adsorbed through cation exchange in these interlayer spaces (Figure 9).

Although the system released more drugs in simulated gastric (pH 1.2) than intestinal (pH 7.4) fluid, it had stronger antibacterial action against *E. coli* and *S. aureus* at pH 7.4. The desorbed tetracycline produced a combination with montmorillonite-free ions, reducing the system’s antibacterial activity at an acidic pH [307].

#### 4.4.2. Enzyme-Responsive Delivery System

A new class of liposomes capable of environment-responsive drug delivery has been created by adsorbing tiny, charged nanoparticles onto liposome surfaces. To do this, we created a liposome formulation that is sensitive to bacteria-secreted phospholipase A2 (PLA2) and chitosan-modified gold nanoparticles (AuChi). The resulting AuChi-stabilized liposomes had no fusion activity and minimal drug leakage. By adding quinacrine dihydrochloride, a PLA2 inhibitor, AuChi-liposomes released their encapsulated payloads when exposed to either isolated PLA2 enzyme or *H. pylori*-produced PLA2. AuChi-liposomes with doxycycline substantially reduced *H. pylori* growth. Enzymes or bacteria present at the infection site stimulated the medication release, allowing for clever “on-demand” antibiotic delivery. The present liposomal delivery technology has vast application potential for tissue microenvironment-responsive medication delivery [308]. Due to their vast surface area and capacity to functionalize their surface via ion exchange, nanosheets are an alternative nanocarrier of interest [309].

Using an emulsification-crosslinking process, a group of scientists developed a novel method of formulating microspheres loaded with Cefuroxime sodium. The microspheres contained admixtures of gelatin and porcine mucin. The results demonstrated that microspheres composed of equal parts of gelatin and mucin exhibited high entrapment effectiveness, which in turn resulted in a high release (up to 85%) and a high bioavailability of the incorporated drug. High drug loading efficiency was also observed in formulations based on different proportions of gelatin and mucin, which led to high drug release in simulated intestinal fluid (SIF) within 3 h. Rapid drug release was noted across all formulations, typically following a biphasic pattern. The mean AUC was found to vary depending on the formulation, ranging from 168 ± 1.93 µg·h/mL for the control to 262 ± 3.47 µg·h/mL for microspheres based on gelatine alone and 328 ± 2.55 µg·h/mL for microspheres based on an equivalent ratio of gelatine and mucin. Cefuroxime sodium’s release and bioavailability in the intestines are improved by S-mucin’s presence in the microspheres. These properties could be utilized in the development of a rectal delivery system [310].

#### 4.4.3. Temperature-Responsive Delivery System

Research in this area is limited, so the study conducted by Heffernan et al. set out to assess the efficacy of a combination of tobramycin and vancomycin delivered locally via temperature-responsive hydrogels in avoiding infection in a rat model of intra-abdominal infection (IAI). In this study, rats were injected intraperitoneally with *E. coli*, then given either no treatment, subcutaneous cefoxitin, or local administration from hydrogels containing vancomycin, tobramycin, or both antimicrobials. It showed that only the hydrogel containing tobramycin and vancomycin significantly improved the infection-free rate compared to no treatment *(E. coli*: 13/17, *p* < 0.0001; cecal contents: 11/17, *p* = 0.0013; and cecal contents + *E. coli*: 15/19, *p* < 0.0001). Furthermore, there was no in vitro synergy or antagonism between tobramycin and vancomycin against clinical isolates. Tobramycin and vancomycin can be locally delivered from temperature-responsive hydrogels, providing long-lasting, high antibacterial concentrations and a wide area of coverage, both of which may be useful in preventing IAIs [311].

#### 4.4.4. Light-Responsive Delivery System

Gold nanoparticles have clinical translation potential. Gold nanoparticles are easily synthesized and biocompatible [312] Few studies have linked photothermal antibacterial treatment in vivo. Physical photothermal action based on gold nanoparticles can prevent antibiotic resistance in clinical settings. Gold nanostars (GNS) have greater photothermal therapeutic potential than other GNPs due to their higher absorption-to-scattering ratio in the near-infrared (NIR) spectral range, more “hotspots” in the branch tip, and multiple sharp edges for heat generation. Their surfactant-free synthesis also makes them biologically safe [313,314].

**Figure 10:**
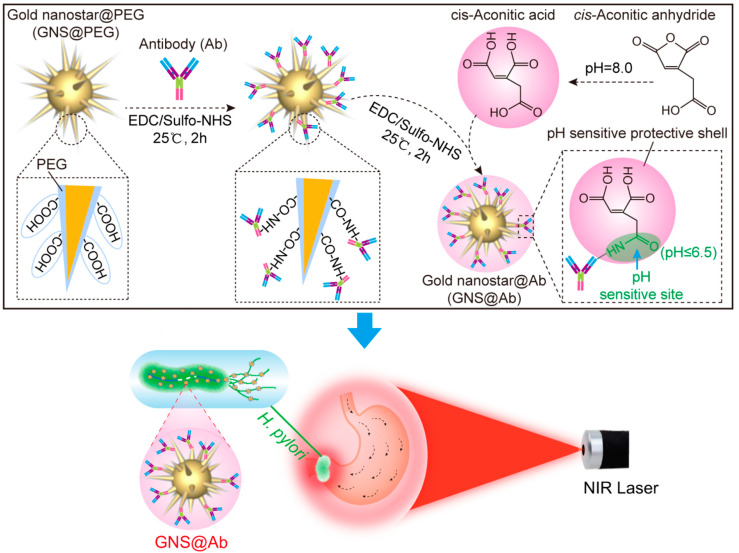
Schematic preparation of pH sensitive GNS@Ab and application for targeted imaging and photothermal therapy of *H. pylori* by in vivo evaluation to treat antibiotic resistance. Reprinted with permission from [313]. Copyright 2019 Elsevier Inc.

Gold nanoparticles are also employed to kill *H. pylori*. Gold nanoparticles destroy H. pylori by disrupting its membrane. Gold nanoparticles enter cells and create ROS, which kill *H. pylori* by disrupting its metabolism. pH-sensitive acid-sensitive cis-aconitic anhydride-modified anti-*H. pylori*-conjugated gold nanostars (GNS@Ab) has been developed. Near-infrared laser photothermal treatment lowered *H. pylori* resistance and even eliminated clinical patient strains. (Figure 10). After *H. pylori* is eradicated in vivo, most GNS@Abs are removed without altering the gut-microbiota balance [313].

### 4.5. Urinary Tract Infection (UTI)

#### 4.5.1. pH-Responsive Delivery System

Microenvironment-targeted antimicrobial administration targets infections and reduces treatment resistance. Acid-responsive materials have been widely studied to cure lactic acid-producing bacterial infections for decades. However, materials can respond to an alkaline bacterial microenvironment, such as chronic wound infections (pH 7.2–8.9), pancreatic infections (7.5–8.5), and catheter-associated urinary tract infections (pH 7.0–8.5) [315,316]. Designing pH-triggered antimicrobial compounds can focus on infected tissue pH. Chen et al. developed an alkaline-responsive antimicrobial hydrogel to fight bacterial infections in diseases with high pH (alkaline). Polydiacetylene-peptide (PDA-Pep) gels have pH-dependent molecular and macromolecular structures. The PDA domain conforms to a blue-to-purple colorimetric transition when the peptide domain is deprotonated by pH increase. Deprotonation causes a gel-to-sol macroscopic phase shift of the fiber network in PDA-Pep hydrogels, which selectively release an antimicrobial drug into the infection site to kill bacteria. The PDA-Pep hydrogels’ pH-sensing and alkaline-triggered antibiotic showed antimicrobial delivery capabilities by using porcine as an animal model. This work sets the groundwork for multifunctional alkaline-responsive materials that can encapsulate numerous small molecule or macromolecular therapies for synergistic biological effects against a wide spectrum of multidrug-resistant diseases [316].

#### 4.5.2. Enzyme-Responsive Delivery System

**Figure 11:**
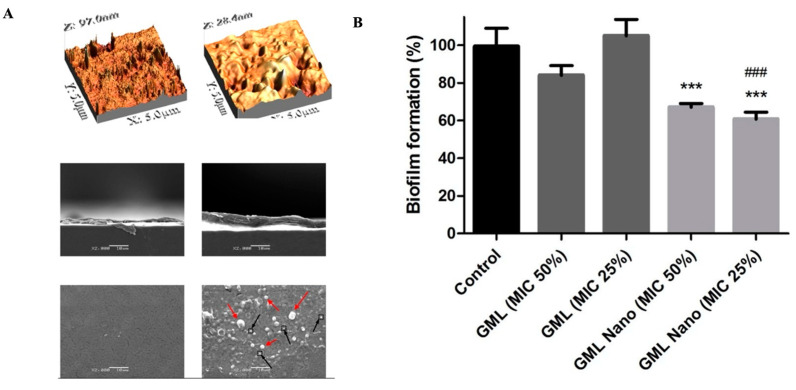
(**A**) AFM images (5 × 5 μm^2^) of (HA/AC_sol_)_10_ and (HA/AC_NSs_)_10_ were deposited onto silicone strips. Followed by a cross-section and top view of the same samples taken with SEM. Reprinted with permission from [320]. Copyright 2016 Elsevier Inc. (**B**) The effectiveness of GML and GML Nano in inhibiting biofilms at 25 and 50 percent of the MIC concentrations. An analysis of variance (ANOVA) followed by Tukey test was used considering values *p* < 0.001 (***) statistically significant comparing with Control and statistically different for *p* < 0.001 (###) comparing with GML in equal concentration. Data was expressed as the Mean ± Standard deviation. Reprinted with permission from [321]. Copyright 2019 Elsevier Inc.

In the search for new treatment strategies for UTIs, they are considered primarily as nanocarriers [317,318]. The production of pathogen biofilms could be inhibited by the presence of nanocarriers. Antibacterial aminocellulose nanospheres (ACNSs) were added to a polydimethylsiloxane (PDS) urinary catheter to reduce E. coli-associated biofilm formation by 80% [319]. Urinary catheters has been tested with ACNSs and HA polyanion to build a layer-by-layer construct on silicone surfaces against Pseudomonas aeruginosa biofilm. The results showed that a 5-layer structure reduced bacteria clusters. No evidence of bacterial colonization was seen in the 10-layer build. AFM and SEM were used to examine the 10-bilayered coatings’ surface topography and morphology. These two coatings revealed a variety of irregularities on their surfaces (Figure 11A). When it comes to surface roughness (R), it was found that the ACsol coating had an R value of 7.7 nanometers, whereas that of the ACNSs had an R value of 3.0 nm. The ACNSs coating has a smoother profile than the ACsol one. Cross-sectional photographs of the coatings show an uneven surface structure, but also total coverage by either (HA/ACsol)10 or (HA/ACNSs)10 after 10 depositions (Figure 11B). A comparison of the dry thicknesses of (HA/ACsol)10 and (HA/ACNSs)10 shows that the ACNSs coating is thicker than the (HA/ACsol)10 coating. Observing the coatings from the top offered further information about their structure because the polycations formed their topmost layer (Figure 11A). Because of the presence of nanospheres on top of them, (HA/ACNSs)10 appeared undamaged (black arrows in Figure 11A) [320].

Glycerol monolaurate (GML) was found to eradicate antibiofilm activity against *Pseudomonas aeruginosa* (Figure 11B). GML is an emulsifier having antibacterial effects against viruses and bacteria [321]. Gold nanoparticles containing chlorhexidine (Au-CHX NPs) were also put to the test against twenty isolates of *K. pneumoniae* from UTI patients and one control strain for antibacterial and antibiofilm activity by the authors (ATCC 13882). All clinical isolates required between 100 and 200 mM to inhibit *K. pneumoniae* ATCC 13882. Clinical isolates’ biofilms were decreased by approximately 90% after exposure to 100 times the concentration required to disrupt the *K. pneumoniae* ATCC 13,882 strain’s biofilm. All isolates’ preformed biofilms were disrupted by 100 mM Au-CHX NPs [322].

### 4.6. Lymphatic Infections

#### 4.6.1. pH-Responsive Delivery System

The lymphatic system allows these large molecules to return to the bloodstream. Due to the unique anatomy of the lymphatic system, drug localization has been difficult [323]. The improved drug absorption and bioavailability of tobramycin-loaded SLN after duodenal administration was attributed to the favored transmucosal transport of SLN to the lymph over the blood [324,325]. Tobramycin-loaded SLN administered duodenally improved drug absorption and bioavailability due to transmucosal transfer to the lymph rather than the circulation [324]. Idarubicin-loaded SLN delivered duodenally increased the drug bioavailability in the same research [326]. Cyclosporine A-loaded dextran acetate particles tagged with 99 mTc were produced following by subcutaneous injection into the footpads of rats; these particles slowly dispersed cyclosporine A throughout the lymph nodes [323].

Development of biodegradable polylactic acid cylinder to target lesions with bleomycin outperformed bleomycin and no therapy [327]. A biodegradable colloidal particulate-based nanocarrier system targeted thoracic lymphatics and lymph nodes to treat lesions. After intrapleural implantation in rats, several nano- and microparticles of charcoal, polystyrene, and poly(lactide-co-glycolide) showed lymphatic absorption after 3 h [328]. In a transplanted tumor model in rats, a comparison of silica particle-based lymphatic drug delivery system of bleomycin to a free bleomycin solution showed that silica particle-adsorbed bleomycin inhibited tumor development and lymph node metastasis more than free bleomycin [323].

#### 4.6.2. Enzyme-Responsive Delivery System

**Figure 12:**
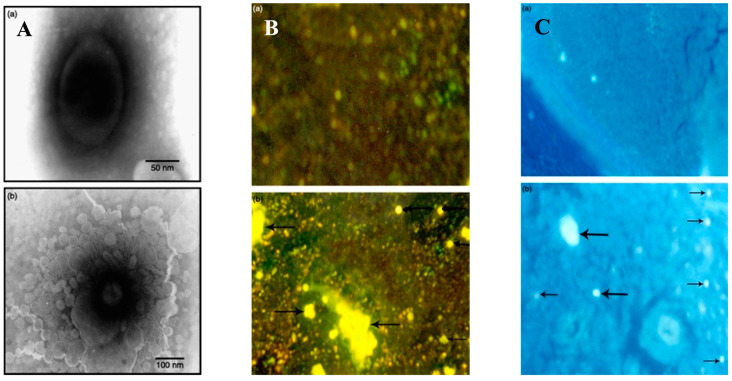
(**A**) TEM photomicrograph of SE liposomes formulation (Man-Lip) at (**a**) 80,000 and (**b**) 120,000. (**B**) Fluorescence photomicrograph of spleen tissue administered with (**a**) drug solution and (**b**) optimized SE liposomes formulation (Man-Lip). Arrows indicate locations of Man-Lip in spleen tissue. (**C**) Fluorescence of photomicrograph of lymph node tissue administered with (**a**) plain drug solution, (**b**) optimized SE liposomes formulation (Man-Lip). The arrows show where the Man-Lip can be found in the lymph node tissue. Copyright 2009. Taylor and Francis journals [329].

Zidovudine (ZDV) as an antiretroviral drug was loaded with surface-engineered liposomes as the subject of a recent study that examined lymphatic delivery (SE liposomes). For lymphatic localization, liposomes were designed to have positive or negative charges and a site-specific mannose (mannose) ligand. There was a use of mannose-terminated SA for the synthesis of positively and negatively charged nanosized SE liposomes (120 ± 10 nm) with ligand-coated SE liposomes (mannose conjugate) (Figure 12A). Liposomes coated with mannose (MAN-Lip) released less of the drug than conventional liposomes in all SE liposome formulations; although, ZDV release was biphasic in these liposomes (*p* < 0.05). The serum-free ZDV concentration was lowered by the SE liposomes, but the spleen and lymph nodes saw an increase (*p* < 0.05). Lymph nodes and the spleen were discovered to contain SE liposomes (Figure 12B,C). Lymphatic enhancement of ZDV targeting by SE liposomes showed promising delivery [329].

When designing a liposomal formulation for the oral administration of a drug, intestinal absorbability and stability are crucial. Liposomal formulation, an aqueous-free drug, and a physical mixing of the drug and empty liposomes were all tested for their ability to improve the oral administration of cefotaxime, a poorly bioavailable hydrophilic medication. The oral bioavailability of the drug was found to be increased by a factor of 2.7 in the liposomal formulation compared to the aqueous dosage and by a factor of 2.3 in the physical mixture. They also took into account that the liposomal formulation results in a marked improvement in the drug’s lymphatic localization in comparison to the other two formulations. To improve the lymphatic transport in the intestinal lymph and the systemic bioavailability of hydrophilic medicines, liposome systems have emerged as promising carriers [330].

### 4.7. CNS Infections

#### 4.7.1. pH-Responsive Delivery System

The blood–brain barrier (BBB) is a major obstacle to the effective delivery of therapeutic agents to the central nervous system (CNS). Because of the BBB, only a few medications are effective in treating brain diseases. In order to increase medication accumulation in lesion areas and limit toxicity in healthy brain tissue, brain-targeted nanocarriers have been developed [331,332,333,334,335]. The biological uses of nanocarriers and their controlled release under varied inputs have been studied [336]. Normal tissue has a pH of around 7.4, but lesions can have a pH as low as 6 [337].

Utilizing the pH properties of the septic environment has been proposed as an effective delivery technique for NAD+ against sepsis. In an effort to enhance the intracellular transport of NAD+, a formulation of pH-responsive, NAD+-loaded, lipid-coated, calcium phosphate (CaP) nanoparticles (NAD+-LP-CaP) and its reduced counterpart, NADH-loaded lipid-coated metal-organic framework (MOF) (NADH-LP-MOF) has been conducted. High amounts of NAD+ may be delivered directly by the nanoplatforms, but only into the slightly acidic cytoplasm. Although the CaP and MOF cores decomposed under acidic conditions in the cytosol, the lipid bilayer coating imparted great serum stability; upon swelling and bursting, the NAD+ was released. The mechanism protected cells from LPS-induced damage by keeping intracellular NAD+ levels stable. The hypothesized mechanism of action is based on the observation that sepsis is characterized by aberrant metabolic activity and elevated energy expenditure due to the combined effects of advanced inflammation and oxidative damage. This causes intracellular NAD+ levels to drop rapidly, leading to cell death. Replenishing intracellular NAD+ hence conserved healthy cells’ energy supply and allowed them to survive sepsis. *Methicillin-resistant Staphylococcus aureus* (MRSA) and *Pseudomonas aeruginosa* were used to produce polymicrobial septicemia, and the effectiveness of this platform in combination with the antibiotic rifampicin (Rif) was evaluated. Tissue histological analysis showed that the combined therapy greatly reduced the bacterial burden and protected against various organs being negatively impacted, in contrast to the free Rif and Rif-LP-CaP, which only produced modest reductions in the bacterial load [338]. This insightful study has the potential to introduce innovative new methods for treating sepsis.

Liposomal drug delivery for meningitis has recently been seen as a new development. Attaching cell-penetrating peptides to the outer membrane of liposomes is one such method. Actively targeted distribution to the cellular membrane is the general mechanism by which peptides are released from the nanostructures. The peptide sequence Tat (YGRKKRRQRRR) enhances the transport of liposomes across the cellular membrane [339]. It has been revealed that Tat-decorated fusogenic liposomes could kill bacteria. The pH changes caused by the fusogenic liposomes destabilizes the bacterial cell. Tat-functionalized fusogenic liposomes were reported to have been generated using the thin-lipid-film rehydration method. In comparison to non-functionalized liposomes, Tat-functionalized liposomes showed much greater bactericidal activity. When compared to non-functionalized liposomes, Tat-decorated liposomes were able to suppress up to 95% of *S. pneumoniae* bacteria. The liposomes with the Tat functionalization were shown to be safe in a cytotoxicity assay using astrocytes and endothelial cells [340]. The blood-brain barrier (BBB) can be crossed by lipid-soluble substances via passive transcellular diffusion, making them a promising drug delivery mechanism for the treatment of brain infections.

#### 4.7.2. Temperature-Responsive Delivery System

The antibacterial activity of PEGylated bacitracin-A nanomicelles was evaluated in a model of pneumococcal meningitis. Combining a *p*-glycoprotein inhibitor (Pluronic^®^ P85 unimers) with a brain-targeted peptide (RVG29) results in a mixed micellar system that improves targeting. The combined micellar system showed both enhanced uptake in brain capillary endothelial cells and good BBB penetration efficiency. Micelles accumulated in brain parenchymal cells through receptor-mediated transcytosis, as revealed by optical imaging. In treating pneumococcal meningitis, animal trials showed encouraging outcomes [341].

#### 4.7.3. Redox-Responsive Delivery System

Fungal infection in CNS can be treated with itraconazole (ITZ) as a commonly prescribed antifungal. Because of its hydrophobic characteristics, inability to cross the BBB, and limited blood circulation stability, ITZ has little effect on cerebral fungal infections. In Jiang’s study, ITZ was loaded into DHA-functionalized nanoscale micelles for successful direct delivery to the target location. DHA-modified micelles are particularly efficient in treating anti-intracranial infections due to their improved blood circulation stability and transport efficiency of ITZ to the brain. Decreased fungal dissemination and increased drug localization in the CNS were also benefits of DHA-PLys(s-s)P micelles’ high stability. DHA-PLys(s-s)P, with high brain permeability and regulated drug deposition, showed a promising approach for CNS drug delivery [342]. Invasive candidiasis in infants commonly manifests as Candidal meningitis. The discovery of antifungal agents with lower side effects and toxicity for children’s medication was discovered by Xu et al. with the development of G3R6TAT. The component was synthesized through the 9-fluorenylmethoxy- carbonyl and CG3R6TAT which was obtained by the synthesizing of cholesteryl chloroformate onto G3R6TAT. This amphiphilic oligopeptide was easily self-assembled into cationic micelles in deionized water at a concentration of ≥31.6 mg/L (i.e., 10.1 μM), and the micelles had a diameter of 177 ± 6 nm with a zeta potential of 55 ± 4 mV. The ability of CG3R6TAT nanoparticles to combat Candida albicans by in vitro testing showed the lowest inhibitory concentration and kill-time curves. Following infection, rabbit models were treated in vivo with either CG3R6TAT nanoparticles (*n* = 6; 0.25 mg/kg/day) or fluconazole (*n* = 6; 100 mg/kg/day) for 11 days, with efficacy assessed by counting yeast in cerebrospinal fluid (CSF), leukocyte concentrations in CSF, and histopathology of the brain parenchyma. At a concentration of 8.1 μmol/l, the nanoparticles completely sterilized C. albicans after 5 h of incubation. Fluconazole or CG3R6TAT nanoparticles effectively reduced the CSF fungal counts and leukocyte concentrations in rabbits compared to untreated control rabbits (*p* < 0.05, ANCOVA). By contrast, the use of fluconazole took 9.7 days to sterilize CSF cultures, while nanoparticles CG3R6TAT took only 8.5 days (*p* > 0.05, vs. control). The degree of histopathology in rabbits was lessened by CG3R6TAT treatment (*p* = 0.001). According to this study, fungal meningitis could be treated using CG3R6TAT nanoparticles proved by in vivo and in vitro evaluation [343].

## 5. Conclusions and Future Perspective

There are various types of polymicrobial diseases that are induced by the growth of polymicrobial in the environment. Antibiotics do not work as effectively on polymicrobial infections because of the issue of resistance and the presence of delivery obstacles to the infected site. Consequently, they necessitate a multifaceted approach in order to alter the course of the disease and prevent antimicrobial drug selection issues. The adoption of new drug delivery technologies using targeted and responsive drug delivery devices with internal (pH, temperature, enzyme, and redox) and external (light, magnetic, and ultrasound) stimuli are compromising. Infection sites have unique microenvironments. Anaerobic glycolysis causes lactic and acetic acid buildup at infection sites that could lower the pH. This can be used for pH-targeted drug release. The development of cationic-charged polymers to release chlorhexidine in acidic oral environments could release chlorhexidine at the infection site, which causes cariogenic biofilm (pH 5.5) to become positively charged. The bacterial infection microenvironment also has a different redox potential than normal cells, which is regulated by NADPH/NADP+ and glutathione levels. This could enable redox-responsive drug delivery devices. In addition, selectively responsive drug delivery devices could use external stimulus by using antibiotic and photothermal (PT) therapy to kill bacteria using near-infrared (NIR) laser-activated targeted drug delivery nano-assemblies. (PTT). Moreover, the ultra-sound release mechanism found that pulsed-laser irradiation of liposomes containing hollow gold nanoshells could also release antibiotics from drug-delivery carriers by co-encapsulating methicillin in the aqueous core of iron oxide poly-mersomes with superparamagnetic iron oxide nanoparticles that can eliminate biofilms. Other examples that are in combination with knowledge of infectious disease pathophysiology, have made the delivery of antimicrobial medications much improved. Therefore, as a potentially effective treatment for diseases brought on by bacterial infections, the use of antimicrobial drugs in the latest trends that is both targeted and responsive, as well as combinatorial, is currently being investigated to achieve its promising treatment. It is then expected that the administration of antimicrobial agents will continue to improve in efficiency, cost, and patient compliance when they are used by ensuring the availability of treatment regimen and its benefits, especially when it’s formed as a targeted and specific medication, as well as the ability to target specific infectious diseases.

Although this approach has made great strides in the pharmaceutical aspects, many limitations still stand in the way. There are a number of challenges that must be taken into account before developing a smart-delivery-based drug delivery system, including biocompatibility, biodegradability, lower toxicity, and safer elimination of smart drug delivery. The administrative challenges associated with polymeric-based smart drug delivery systems are another significant setback in this area. Another factor to think about is the carrier’s size. Future research should investigate and develop drug delivery systems made from new polymeric materials that are biocompatible and non-toxic to address these issues. More extensive studies are still required in this field, including clinical trials, prior to bringing the formulations to the patient’s bedside. Nevertheless, the myriad of studies that were discussed in this review showed that stimuli-responsive delivery is a promising novel approach that may provide a way to treat polymicrobial diseases.

## Figures and Tables

**Table 1:** Empiric recommendation for all ages with pulmonary infection [124].

The Location of Treatment	Causes of Infection	Treatment Options
Inpatient, Non-ICU	*H. influenzae* *S. aureus* *S. pneumoniae*	*β-lactam plus macrolide:* Cefotaxime IV/IM + Azithromycin oral Ceftriaxone IV/IM + Azithromycin oral Ampicillin IV/IM + Azithromycin oral *Alternative to macrolide:* Doxycycline oral *Respiratory fluoroquinolone:* Levofloxacin, Moxifloxacin, or Gemifloxacin
Inpatient, ICU	*Legionella* *H. influenzae* Gram-negative enteric organisms *S. pneumoniae* (including drug resistant)	*β-lactam plus macrolide:* Cefotaxime, Ceftriaxone, or Ampicillin-sulbactam IV/IM. *Plus:* Azithromycin or Levofloxacin IV *Penicillin allergy:* levofloxacin + aztreonam IV
Inpatient, ICU	*Methicillin resistant S. aureus* (MRSA)	*β-lactam plus macrolide:* Cefotaxime, Ceftriaxone, or Ampicillin-sulbactam IV/IM. *Plus:* Azithromycin or Levofloxacin IV *Penicillin allergy:* levofloxacin + aztreonam IV * Plus:* Vancomycin IV or Linezolid IV or oral
Inpatient/ICU	*Pseudomonas aeruginosa*, suspected	*Anti-pseudomonal β-lactam:* Piperacillin-tazobactam IV, Cefepime IV, Imipenem IV *Plus, either:* Ciprofloxacin IV, Levofloxacin IV *Penicillin allergy:* Aztreonam IV can be substituted for β-lactam

**Table 2:** Management therapy for specific microorganisms in bacterial meningitis [160,161].

Cause of Infection	Antibiotic Treatment	Duration of Therapy
*Streptococcus pneumoniae Penicillin-*susceptible isolate	Adults: penicillin G or ampicillin IV. Children: penicillin G or ampicillin. Severe penicillin allergy: substitute cephalosporin agent with chloramphenicol.	10–14 days
*Neisseria meningitidis*	Adults: penicillin G IV or ampicillin IV or ceftriaxone IV or cefotaxime IV. Penicillin allergy: substitute cephalosporin agent with chloramphenicol. Children: penicillin G IV. Penicillin allergy: substitute with chloramphenicol.	7 days
*Haemophilus influenza*	Betalactamase positive: ceftriaxone IV or cefotaxime IV Betalactamase negative: Ampicillin IV	7 days
*Pseudomonas aeruginosa*	Ceftazidime IV or cefepime IV plus gentamicin IV	21 days
Group B *Streptococcus* Suspected/empiric	Preterm: ampicillin IV plus cefotaxime. Infants ≤7 days: ampicillin IV plus aminoglycoside Infants >7 days: ampicillin IV plus an aminoglycoside, Intraventricular treatment not recommended.	14–21 days
Enterobacteriaceae	Ceftriaxone IV or cefotaxime IV plus gentamicin IV	21 days
*Listeria monocytogenes*	Infants ≤7 days: ampicillin IV plus aminoglycoside, Infants >7 days: ampicillin IV plus aminoglycoside. Adults >50, alcoholism, or other risk factors: ampicillin IV plus ceftriaxone IV or cefotaxime IV plus gentamicin IV loading dose, then plus dexamethasone IV Penicillin allergy: trimethoprim/sulfamethoxazole	21 days

**Table 3:** Treatment of bacterial infections on the GI tract [179].

Disease	Pathogen	Treatment
*Bacillus cereus* infection	*Bacillus cereus*	Rehydration and supportive therapy. Antibiotics are usually not needed.
*Campylobacter jejuni* gastroenteritis	*Campylobacter jejuni*	Antibiotics are usually not required, but erythromycin or ciprofloxacin may be used.
*Cholera*	*Vibrio cholera*	Antibiotics are usually not required, but tetracycline, azithromycin, and others may be used.
*Clostridium difficile* infection	*Clostridium difficile*	First, stop the use of antibiotics and provide supportive therapy with electrolyte and fluid replacement. Metronidazole or vancomycin may also be used.
*Clostridium perfringens* gastroenteritis	*Clostridium perfringens* (especially type A)	Rehydration therapy, electrolyte replacement, and intravenous fluids. Antibiotics are not recommended.
*Escherichia coli* (*E. coli*) infection	*Enterotoxigenic E. coli* (ETEC)	Self-limiting; if needed, fluoroquinolones, doxycycline, rifaximin, TMP/SMZ; antibiotic resistance is a problem.
	*Enteroinvasive E. coli* (EIEC)	Supportive therapy only; antibiotics not recommended.
	*Enteropathogenic E. coli* (EPEC)	Self-limiting; if needed, fluoroquinolones, doxycycline, rifaximin (TMP/SMZ); antibiotic resistance is a problem.
	*Enterohemorrhagic E. coli* (EHEC)	Antibiotics are not recommended due to the risk of HUS.
Peptic ulcers	*Helicobacter pylori*	Amoxicillin, clarithromycin, metronidazole, tetracycline, and lansoprazole. Antacids may also be given in combination with antibiotics.
Salmonellosis	*Salmonella enterica*, serotype Enteritides	Oral rehydration therapy. Antibiotics are only recommended for serious cases such as in immunocompromised patients; fluoroquinolones, third-generation cephalosporins, and ampicillin are recommended.
Shigella dysentry	*Shigella dysenteriae*, *S. flexneri*, *S. boydii*, and *S. sonnei*	Ciprofloxacin and azithromycin.
Staphylococcal food poisoning	*Staphylococcus aureus*	Without treatment this condition usually heals relatively quickly, within 24 h.
Typhoid fever	*S. enterica*, serotype Typhi or Paratyhphi	Fluoroquinolones, ceftriaxone, and azithromycin. Preventive vaccine available.
Yersinia infection	*Y. enterocolitica* and *Y. pseudotuberculosis.*	Supportive therapy such as rehydration without antibiotics. Fluoroquinolones, aminoglycosides, doxycycline, and trimethoprim-sulfamethoxazole may be used for systemic infections.

**Table 4:** Treatment of viral infections of the GI tract [179].

Disease	Pathogen	Treatment
Astrovirus gastroenteritis	Astrovirus	Supportive therapy such as rehydration and electrolyte replacement.
Norovirus gastroenteritis	Norovirus	Supportive therapy.
Rotavirus gastroenteritis	Rotavirus	Supportive therapy and vaccinations for babies.

**Table 5:** Treatment of protozoal infections of the GI tract [179].

Disease	Pathogen	Treatment
Amoebiasis (amoebic dysentery)	*Entamoeba histolytica*	Metronidazole, tinidazole, diloxanide furoate, iodoquinol, and paromomycin.
Cryptosporidiosis	*Cryptosporidium parvum*, *Cryptosporidium hominis*	Oral rehydration therapy and anti-parasitic drugs (nitazoxanide, azithromycin, and paromomycin).
Cyclosporiasis	*Cyclospora cayetanensis*	Trimethoprim-sulfamethoxazole.
Giardiasis	Giardia lamblia	Metronidazole and tinidazole.

## Data Availability

Available data are presented in the manuscript.
